# The potentiality of nodule-inhabiting fungi to control the growth of *Fusarium oxysporum*

**DOI:** 10.1007/s11274-026-04893-2

**Published:** 2026-04-18

**Authors:** Aya A. Elemam, Amr M. Mowafy, Yasmin M. Heikal, Fatma F. Migahed

**Affiliations:** 1 Botany Department, Faculty of Science, Mansoura University, Mansoura, 35516 Egypt; 2 Department of Biological Sciences, Faculty of Science, New Mansoura University, New Mansoura City, Egypt

**Keywords:** Bio-control, *Fusarium oxysporum* f. sp.* phaseoli*, Nodule-inhabiting fungi, *Phaseolus vulgaris* L., Plant growth promotion, *Talaromyces pinophilus* TP-BAC1

## Abstract

This study explored the isolation and characterization of non-rhizobial endophytic fungi (NREF) from plant root nodules, emphasizing their potential significance in the biological control of *Fusarium oxysporum* f. sp*. phaseoli* (Fop) and in enhancing the growth of *Phaseolus vulgaris* L. Seventeen fungal isolates were obtained, seven of which had substantial antagonistic activity against Fop. Morphological identification identified these isolates as *Aspergillus fumigatus*, *Paecilomyces variotii*, *Talaromyces pinophilus* (three isolates), *Trichoderma harzianum*, and *Trichoderma longibrachiatum*. All selected isolates were assessed for plant growth-promoting (PGP) characteristics, encompassing indole acetic acid (IAA) and gibberellic acid (GA_3_) production, phosphate and zinc solubilization, siderophore and ammonia (NH_4_) generation, and hydrolytic enzyme activities. *Talaromyces pinophilus* TP-BAC1 had the highest performance in most traits, with an inhibition zone of 22.5 mm. In a pot experiment, this isolate significantly (P ≤ 0.05) decreased the disease index from 81.7% in the *Fusarium* treatment to 15.72% in bean plants, resulting in a protection efficiency of 80.75%. Additionally, TP-BAC1 treatment increased the amount of chlorophyll, antioxidants, and membrane stability, which helped bean plants deal with stress caused by *Fusarium*. The results underscore the significant potential of *Talaromyces pinophilus* TP-BAC1 as a sustainable biocontrol agent and biofertilizer in bean cultivation, providing an ecologically friendly alternative to chemical fungicides.

## Introduction

The common bean (*Phaseolus vulgaris* L.) is an essential leguminous crop cultivated globally. It is a vital source of fiber, protein, vitamins, and several minerals for human nutrition (Suárez-Martínez et al. 2016). The cultivation area of common beans is second behind that of soybeans and peanuts. The common bean is extensively grown as an essential food source globally, particularly in Africa and South America (Assefa et al. 2019). In Egypt, among the most significant vegetable crops for both domestic expenditure and export is the common bean. The statistics of the Egyptian Ministry of Agriculture in 2022 showed that, with an average yield of 3.18 Mg ha⁻^1^, the 116.76 ha of green common bean production produced roughly 371.41 Mg (MALR, 2022).

The cultivation of common beans is hindered by various ecological conditions, including biological and environmental stresses (Pathania et al. 2014). *Fusarium* wilt is a significant fungal disease of common bean, a soil-inhabiting pathogen impacting plentiful harvests worldwide. All *Fusarium* species responsible for vascular wilting are classified as *Fusarium oxysporum* (Pereira et al. 2009), encompassing several forma speciales based on pathogenic criteria. MycoBank enumerates 127 formae speciales of *F. oxysporum* (Jha et al. 2020). They contribute to the spread of the disease in more than 100 plant species (Pantelides et al. 2013). *Fusarium oxysporum* f. sp. *phaseoli* is categorized within the Ascomycota division of imperfect fungus, specifically in the class Sordariomycetes, order Hypocreomycetidae, family Nectriaceae, and species *F. oxysporum* (Lombard et al. 2019). Fusarial wilt disease is caused by *F. oxysporum* that is Schlechtend. Fr f. sp. *phaseoli* (Fop) J.B. Kendrich and W.C. Snyder, and was detected and classified in the majority of bean cultivation areas globally (Rampersad 2020). Fop, the disease-causing organism of *Fusarium* wilt in common bean, is presently regarded as one of the foremost prevalent diseases affecting beans (Paulino et al. 2020). *F. oxysporum* sp. *phaseoli* is capable of invading intact root tissue as well as more mature regions of the root and hypocotyl tissues, typically by means of injuries or normal openings. Fop enters plants via the root system and proliferates the xylem, resulting in wilting, vascular browning, yellowing, stunting, and early**-**stage plant mortality (Sasseron et al. 2020). In resistant varieties, symptoms are diminished or less severe due to the secretion of a blocking material in xylem channels (Pereira et al. 2013). Plants have active adaptive defense system for self-protection from invasive diseases such as fungi, bacteria, viruses, and oomycetes (Bigeard et al. 2015). Plants initiate local defenses that elicit a subsequent immunological response termed systemic acquired resistance. SAR confers long-term resistance to the non**-**infected distant sites through the activation of several pathogenesis-related genes (Naz et al. 2024). Numerous chemicals have been suggested as mobile signals that induce SAR (Klessig et al. 2018). To mitigate the effects of these diseases, there is growing interest in understanding the complex communications among pathogens and the plant-related microbiota, including fungi with growth-promoting activity (PGPF) found in root nodules. Nodule-borne fungi (NBF), often overlooked in symbiotic nitrogen-fixation studies, are increasingly predictable for their function in modulating stimulation of plant development and pathogen resistance in legumes. These non-rhizobial symbionts can enhance nodulation and biological nitrogen fixation, partly through modulation of host signaling pathways and improved nutrient acquisition (Debnath et al. 2023). Many isolates exhibit multiple growth-enhancing characteristics, including IAA production, phosphate, potassium solubilization, siderophore production, and ACC-deaminase activity, which collectively stimulate root development, increase nutrient uptake, and alleviate stress ethylene under abiotic constraints (Airin et al. 2023). In addition, several nodule endophytes demonstrate antagonistic activity against phytopathogens through antibiosis and induced systemic resistance, thereby protecting root systems, sustaining yield stability, and becoming valuable bioinoculants for sustainable agriculture (Hamane et al. 2023). Recent studies indicate a significant diversity of non-rhizobial endophytes coexisting alongside rhizobia in the root nodules of Vigna species, without adversely affecting plant health (da Silva et al. 2021). The presence of fungi within nodules provides a valuable model for studying early-stage plant-pathogen interactions. For instance, species such as *Fusarium oxysporum* and *Alternaria alternata* have been reported to colonize legume root tissues and even root nodules, where they can behave either as endophytes or as pathogens. Their lifestyle often depends on host genotype and prevailing environmental conditions, with some strains establishing symptomless associations that may enhance plant fitness, while others trigger disease when the host is stressed or when resistance mechanisms are compromised (Hnini and Aurag 2024). Consequently, employing specific microorganisms to protect crops from soilborne diseases presents an attractive substitute to conventional pesticides, which exhibit limited efficacy and a significant risk of environmental contamination (Heydari and Pessarakli 2010).

This study aimed to isolate and characterize endophytic fungi from plant root nodules, studying their suppressive potential against *Fusarium oxysporum* f. sp. *phaseoli*, and investigating their role in promoting plant development. Across the recovered isolates, *Talaromyces pinophilus* TP-BAC1 effectively suppressed *Fusarium* infection and enhanced plant growth, as evidenced by improved morphological and physiological traits. These results present *Talaromyces pinophilus* TP-BAC1 as a potential plant growth promoter with a dual role.

## Materials and methods

### Materials

The laboratory-grade chemicals utilized in this experiment were supplied by local companies, namely El-Gomhoria Company, Egypt.

### Isolation of nodule-borne fungi

Root nodules from *Medicago sativa* L., *Phaseolus vulgaris* L., and *Vicia faba* L. were collected from the experimental field at Mansoura University's Faculty of Agriculture, after securing permission from the field's administration. The plants were formally identified by Prof. Dr. Ibrahim Mashaly, Professor of Plant Ecology and Flora in the Botany Department at Mansoura University's Faculty of Science, Egypt, following Boulos (Boulos 2008), by comparing them to reference specimens in the Botany Department's Herbarium. Voucher specimens were deposited in the University herbarium with the numbers: *Medicago sativa* L. (Voucher No. MS-002), *Phaseolus vulgaris* L. (Voucher No. PV-005), and *Vicia faba* L. (Voucher No. VF-009). All procedures were complied with institutional and local regulations.

Viable pink and healthy nodules were collected and subjected to surface sterilization sequentially with 70% ethyl alcohol for 30 s, subsequently treated with 2% sodium hypochlorite for 3 min, and which three times with sterilized distilled water. Sterilized nodules were subsequently aseptically macerated and inoculated onto Potato dextrose agar media (PDA) enriched with chloramphenicol (500 mg L⁻^1^). Plates were maintained at 28 °C for 5 days of incubation, after which emerging fungal colonies were obtained and stored on PDA slants using standard subculturing for subsequent investigations (Somasegaran and Hoben 2012).

### Morphological identification of nodule-borne fungi

Identification of fungal isolates relied on observations of their macroscopic and microscopic traits, encompassing colony growth, hyphal structure, pigmentation, mycelial texture, sporulation, and conidial morphology, as they were observed after 3, 6, 9, and 12 days (on PDA) and matched with standard taxonomic keys (Barnett and Hunter 1972; Devi and Prabakaran 2014). Microscopic examination was performed after lactophenol cotton blue staining using bright-field and phase-contrast microscopy at 400 × magnification (Khalil et al. 2021).

### Characterization of plant growth-promoting attributes in fungal isolates

#### IAA metabolite production

Khalil et al. (2021) assessed IAA production by inoculating fungal endophytes isolated from nodules into potato dextrose broth (PDB) augmented with 1.02 g L⁻^1^ of 1-tryptophan, subsequently incubated at 28 °C for 5 days in an orbital shaking incubator set at 150 rpm. Following centrifugation of the culture at 7000 rpm for 3 min, the assay was performed by combining 1 mL of culture supernatant with 2 mL of Salkowski reagent prepared from 60% perchloric acid and 3 mL of 0.5 M FeCl_₃_ solution. The evaluation of IAA content can be conducted by measuring the absorption of pink color at 530 nm. The concentration of IAA was evaluated with a standard curve of indole acetic acid ranging from 10 to 100 µg ml⁻^1^.

### GA₃ production assay

GA_3_ production was quantified after inoculating NBF isolates in PDB broth medium, which was maintained at 28 °C with continuous shaking at 150 rpm for 14 days. After centrifuging at 5000 rpm, 15 mL of the resulting supernatant was combined with 2 mL of zinc acetate reagent and 2 mL of 10.6% potassium ferrocyanide, and the mixture was subsequently centrifuged at low speed for 2 min. The collected supernatant (15 mL) was reacted with 5 mL of 30% HCl and incubated at 25 °C for 75 min. Absorbance was read at 254 nm, against 5% HCl blank. Gibberellin levels (100–1000 μg mL⁻^1^) were quantified using gibberellic acid for standard curve preparation, following minor modifications to the method of (Borrow et al. 1955).

### Ammonia production assay

Ammonia production was assessed by inoculating 10 mL of peptone water broth with NBF isolates, followed by incubation at 28 °C for 5 days. After incubation, 1mL of Nessler’s reagent was added to each tube. The intensity of the color change, from light yellow to brown, corresponds to the amount of ammonia produced. Absorbance was measured at 450 nm using a spectrophotometer. Ammonium sulfate standards (0.5–25 mg L^−1^) were used to generate a calibration curve for determining ammonia concentration in the samples (Khalil et al. 2021).

### Nitrogen-fixing potential

Nitrogen fixation was assessed as a preliminary qualitative screening to evaluate the potential ability of the isolates to grow under nitrogen-limited conditions. Nitrogen fixation potential of the NBF was assessed on a nitrogen-free medium consisting of (g/L): 5.0 glucose, 0.2 KH₂PO₄, 0.2 MgSO₄0.7H₂O, 0.2 NaCl, 0.1 CaSO₄0.2H₂O, 5 CaCO₃, and 15 agar, with the pH maintained between 7.0 and 7.2. Nodules-borne fungal isolates were incubated on nitrogen-free medium at 28 °C for 7 days, and colony proliferation was considered evidence of nitrogen fixation ability (Fu et al. 2022).

### Phosphate-solubilizing activity

Phosphate solubilization by fungal isolates from nodules was evaluated on NBRIP medium (Liu et al. 2015) containing (g/L): 10 Glucose, 5 Ca₃(PO₄), 5 MgCl₂0.6H₂O, 0.25 MgSO₄0.7H₂O, 0.2 KCl, 0.1 (NH₄)₂SO₄, and 15 agar. The medium pH was adjusted to 7.0 before sterilization. Nodule**-**associated fungal endophytes were incubated on NBRIP agar medium at 28 °C for 7 days to assess phosphate solubilization, indicated by the formation of clear zones around the colonies. Quantitative estimation was performed using NBRIP broth cultures, whose supernatants obtained after centrifugation at 10,000 rpm for 10 min, were analyzed by the molybdenum antimony colorimetric method (Lü et al. 2023). Potassium dihydrogen phosphate was used to prepare the standard curve.

### Zinc solubilization

Zinc solubilizing potential of nodule-associated fungal isolates was determined using Mineral Salt Agar (MSA) medium (pH 7.0) supplemented with 0.1% ZnO as an insoluble zinc source (Saravanan et al. 2007). A 6 mm mycelial disc from PDA- growth cultures was inoculated at the center of MSA plates and incubated at 25 ± 1 °C. Three replicates were prepared for each isolate, with uninoculated plates serving as control. Observations of halo zone formation were recorded on days 3, 5, 7, and 10 post-inoculation. Zn solubilization efficiency (SE) was evaluated by calculating the ratio of the clear halo zone diameter to the colony diameter. Quantitative determination of solubilized zinc by NBF was conducted using an Atomic Absorption Spectrophotometer (AAS) (Model 210VGP/Accusys 211, Buck Scientific, USA). The analysis was repeated on days 5, 7, 10, and 15 post-inoculation. Solubilized zinc content was obtained by subtracting the Zn concentration of uninoculated controls from that of inoculated samples and expressed in ppm.

### Siderophore production

Siderophore development was assessed by growing the fungal isolates in iron-deficient Grimm-Allen liquid medium (10⁶ spores/mL, 28 °C, 15 days) (Zhang et al. 2020). Culture filtrates were mixed with Chrome Azurol S (CAS) reagent and shuttle solution, followed by incubation for 10 min at room temperature. The absorbance was then measured at 630 nm. The percentage of siderophore units was calculated using the following formula:

Siderophore % = **(**(Ar-As)/Ar**)** × 100.

Where Ar represents the absorbance of the reference(uninoculated control) and As ​represents the absorbance of the sample.

### Enzymatic activities

Amylase activity of NBF isolates was assessed by inoculating them onto starch agar medium and incubating the plates at 28 °C for 5–7 days. After incubation plates were flooded with iodine solution. The appearance of clear halos around the fungal colonies indicated positive amylase activity (Hankin and Anagnostakis 1975). Cellulase activity of fungal isolates was evaluated by inoculating them onto cellulase agar medium and incubating at 28 °C for 2 −5 days. After incubation plates were flooded with 0.2% aqueous Congo red solution, followed by destaining with 1 M sodium chloride for 15 min. The appearance of clear zones around the colonies indicated cellulase activity, as described by Hankin and Anagnostakis (1975). To assess pectinase production, NBF isolates were grown on pectinase agar medium and at 28 °C for 2–5 days. After incubation plates were overlaid with 1% CTAB solution. The appearance of clear zones surrounding the colonies confirmed pectinase activity in accordance with the described method (Hankin and Anagnostakis 1975).

### Characteristics of NBF isolate extracts

#### Antagonistic activity of crude filtrates

Fungal isolates were grown in PDB and incubated at 30 °C for 5 days under shaking conditions. After incubation, cultures were centrifuged at 20,000 rpm for 10 min at 4 °C, and the resulting supernatant was passed through a 0.22 µm membrane to obtain cell-free filtrates. Crude filtrates were preserved in sterile containers and tested against *Fusarium oxysporum* f. sp. *phaseoli* (Fop). A pathogen disc was inoculated onto the filtrate's surface and incubated at 28 °C for 7 days. Visible pathogen growth signified negative activity, while growth inhibition reflected positive antagonistic potential.

**Antifungal activity of ethyl acetate** (**EtOAc**) **crude filtrates.**

Endophytic fungi were cultivated under submerged fermentation in 250 mL of PDB (in 500 mL flasks) at 28 °C and 150 rpm for 10 days. Secondary metabolites were extracted from the culture filtrates using ultrasonic-assisted ethyl acetate extraction. The antifungal activity of EtOAc extracts was tested against Fop by the agar well diffusion method (Hashem et al. 2022). PDA plates containing 2–4 day-old pathogen culture were prepared, and three wells (5 mm in diameter, 20 mm from the pathogen colony) were filled with 50 µL of extract. Plates were incubated at 28 °C for 3–5 days, and the inhibition zones were measured as an indicator of antifungal activity.

### Estimation of total phenolic and flavonoid contents

Non**-**enzymatic antioxidant compounds in EtOAc extracts were quantified as described by (Kosem et al. 2007). Total phenolics were estimated using the Folin-Ciocalteu reagent at 765 nm and represented as gallic acid equivalents (mg GAE/g DW). Total flavonoids were determined via the aluminum chloride method at 510 nm, and the results were expressed as quercetin equivalents (mg QE/g DW) (Gorinstein et al. 2009).

### Antioxidant activity via DPPH radical scavenging assay

Antioxidant potential of the extracts was determined using the 2,2-diphenyl-1-picrylhydrazyl (DPPH) free radical scavenging assay. Fungal extracts (1 mg/mL) were mixed with 0.5 mmol/L DPPH solution prepared in methanol and incubated at room temperature in the dark for 30 min. The absorbance of each reaction mixture was measured spectrophotometrically at 517 nm. The percentage of DPPH radical scavenging activity was calculated using the following formula:

$$\mathbf{\%}\mathbf{I}\mathbf{n}\mathbf{h}\mathbf{i}\mathbf{b}\mathbf{i}\mathbf{t}\mathbf{i}\mathbf{o}\mathbf{n}=\left(\left(\mathbf{A}\mathbf{i}-\mathbf{A}\mathbf{t}\right)/\mathbf{A}\mathbf{i}\right)\times 100$$

where Ai represents the absorbance of the control (DPPH solution without extract), and At is the absorbance of the sample containing extracts.

### Greenhouse evaluation of nodule-borne fungal (NBF) isolates on *Fusarium*-infected *Phaseolus vulgaris* L.

The pot experiment was conducted in the greenhouse of the Faculty of Agriculture, Mansoura University, Egypt, under natural light conditions (12 h day/12 h night) at 28 °C and controlled humidity. Autoclaved soil mixtures (clay: sand, 2:1) were prepared, with 3 kg of soil placed in each plastic pot. Homogeneous and viable seeds of the *Phaseolus vulgaris* L., Nebraska bean cultivar obtained from the Field Crops Research Institute, Sakha Agricultural Research Station, Agricultural Research Center (ARC), Sakha, Kafr El-Sheikh, Egypt, were surface sterilized using 5% NaClO for 3 min, followed by thoroughly rinsing with sterile distilled water. The seeds were divided into four experimental groups: (i) control, soaked in sterile distilled water; (ii) seeds primed with NBF spore suspension (1 × 10⁶ spores/mL) and subsequently challenged with *F. oxysporum* (1 × 10⁶ spores/mL); (iii) seeds inoculated only with *F. oxysporum* (1 × 10⁶ spores/mL) two weeks after NBF application; and (iv) seeds treated with NBF spore suspension (1 × 10⁶ spores/mL) only to evaluate its growth-promoting effect in the absence of pathogen challenge. For each treatment, five seeds were sown per pot at a depth of 1 cm beneath the soil surface.

### Disease severity and protection estimation

Disease symptoms appeared 45 days after inoculation. The intensity of infection and protective potential of nodule-borne fungal isolates were assessed using a 0—4 disease severity scale, where: 0 = no visible symptoms; 1 = slight yellowing of lower leaves; 2 = significant yellowing; 3 = complete wilting and browning of vascular bands; 4 = severe stunting and plant death (Abdelaziz et al. 2022). The percent disease index (PDI) was calculated using the following formulas:

$$\mathrm{PDI}=\frac{(1n1+2n2+3n3+4n4)}{4nt}\times 100$$

where n_1_–n_4_ represent the number of plants in each category and nt denotes the total number of plants. The percent of protection was determined as:

$$\mathrm{Protection}\left(\%\right)=\frac{A-B}{A}\times 100$$

where, A is the PDI in infected control plants, B is the PDI in treated infected plants.

### Plant morphological parameters measurement

Measurements were recorded for root length, shoot length, root fresh and dry weights, shoot fresh and dry weight, and the number of leaves of 45-day-old plants. Photosynthetic pigments were extracted from 0.1 g of fresh leaf tissue using 5 mL of DMSO at room temperature and left for 24 h. The extracts were then filtered, and the final volume was adjusted to 10 mL. Absorbance was recorded at 470, 644, and 662 nm, and the concentrations of chlorophyll a, chlorophyll b, and carotenoids were calculated using standard equations (Adhikari et al. 2020).

$$\begin{array}{c}\mathrm{Chl}.\text{a }= 12.7\left({\mathrm{O}.\mathrm{D}}_{662}\right)-2. 69 \left({\mathrm{O}.\mathrm{D}}_{644}\right)=\text{ mg}/\mathrm{mL}\\ \mathrm{Chl}.\text{a }= 22.9\left({\mathrm{O}.\mathrm{D}}_{664}\right)-2. 69 \left({\mathrm{O}.\mathrm{D}}_{662}\right)=\text{ mg}/\mathrm{mL}\\ \mathrm{Carotenoids}=\left[\left({\mathrm{O}.\mathrm{D}}_{470}\right)-1.28\left(\mathrm{Chl}.\mathrm{a}\right)+5.67\left(\mathrm{Chl}.\mathrm{b}\right)\right]/\left(256\times 0.906\right)=\mathrm{mg}/\mathrm{mL}\end{array}$$

### Plants secondary metabolites

Total phenolic compounds were extracted from 2 g of powdered dry tissue using 50% methanol and incubated at 37 °C for one week. Subsequently, 1 mL of extract was mixed with Folin**-**Ciocalteu reagent and sodium carbonate, followed by incubation in the dark for 90 min. The absorbance was then recorded at 760 nm. The results were expressed as micrograms of gallic acid equivalents (GAE) per gram of dry weight (Zeitoun et al. 2017). For total flavonoid determination, 1 mL of the methanolic extract was sequentially mixed with NaNO₂, AlCl₃, and NaOH, and the final volume was adjusted to 10 mL. Absorbance was measured at 500 nm, and flavonoid contents were expressed as milligrams of quercetin equivalents (QE) per gram of dry weight (De Souza et al. 2018).

### Membrane and stress indices in plant leaves

The Membrane Injury Index (MII) was determined by incubating fresh leaf samples (0.1 g) in 10 mL of distilled water at 40 °C for 30 min (EC1) and subsequently at 100 °C for 15 min (EC2). Electrical conductivity was measured after each temperature treatment. The MII was calculated as (EC1/EC2) × 100 (Kocheva et al. 2014). The Membrane Stability Index (MSI) was calculated as 100 minus MII (Mickky et al. 2019).

The concentration of hydrogen peroxide (H₂O₂) was determined from the supernatant of fresh leaf tissue (0.2 g) homogenized in 5 mL of 0.1% trichloroacetic acid (TCA). The homogenate was centrifuged, and the resulting supernatant was combined with potassium iodide (KI) and potassium phosphate buffer. The absorbance of the reaction mixture was measured at 390 nm, and the H₂O₂ content was ascertained using a standard curve as described by Alexieva et al. (2001).

Lipid peroxidation was assessed by estimating malondialdehyde (MDA) content. Fresh tissue (1 g) was homogenized in 0.1% TCA and centrifuged at 10,000 × g for 5 min. To 1 mL of the resulting supernatant, 4 mL of 20% (w/v) TCA with 0.5% (w/v) thiobarbituric acid (TBA) was added. The reaction mixture was incubated at 95 °C for 30 min, then immediately cooled in an ice bath. After cooling, the samples were centrifuged again at 10,000 × g for 15 min, and the absorbance of the supernatant was recorded at 532 nm (corrected at 600 nm). The MDA concentration was quantified using an extinction coefficient of 155 mM⁻^1^ cm⁻^1^ and expressed as µmol MDA/g of fresh weight (Heath and Packer 1968).

### Molecular identification of fungal isolates

#### DNA extraction and PCR amplification

Genomic DNA was extracted from the fungi using a Zymo Fungal DNA extraction kit, derived from a one-week-old PDA culture. The purity and concentration of the DNA extracted were evaluated using a Nanodrop Spectrophotometer (ND 1000, Thermo Scientific, USA). All samples displayed a DNA yield between 5 and 25 ng, with the extracted DNA showing high purity, as evidenced by an A260/A280 ratio of 1.60 to 1.80. Internal transcribed spacer (ITS) region of the ribosomal DNA was amplified using universal primers designed with Primer3 software version 4.1.0 (https://primer3. Org/): ITS1 (5'—TCC GTA GGT GAA CCT GCG G—3'), and ITS4 (5'—TCC TCC GCT TAT TGA TAT GC—3'). Amplification was conducted with the ABI 9700 thermal cycler. The polymerase chain reaction (PCR) mixtures were developed utilizing Solgent EF-Taq as detailed below: 10X EF-Taq buffer solution, 2.5 µL of 10 mM dNTP, 0.5 µL of primer (F-10p), 1.0 µL of primer (R-10p), 0.25 µL of EF-Taq (2.5U), 1.0 µL of template, and distilled water to a final volume of 25 µL. The amplification was conducted under the subsequent PCR reaction conditions: One round of amplification was conducted, comprising denaturation at 95 °C for 15 min, succeeded by 30 cycles of denaturation at 95 °C for 20 s, annealing at 50 °C for 40 s, and extension at 72 °C for 1 min, culminating in a final extension at 72 °C for 5 min (Singh et al. 2020). The PCR products were subsequently purified using the SolGent PCR Purification Kit-Ultra (SolGent, Daejeon, South Korea) before sequencing.

### Sequencing of ITS region of rRNA gene

The purified PCR products were verified using a size marker via electrophoresis on a 1% agarose gel. The bands were subsequently eluted and sequenced. Each sample was sequenced in both the sense and antisense directions utilizing identical primers and ddNTPs (Big Dye). Contigs were generated from the sequencing data with the CLCBio Main Workbench software. The acquired sequences were subsequently examined with BLAST from the National Center for Biotechnology Information (NCBI) website. The sequence acquired, along with those sourced from the GenBank database (http://www.ncbi.nlm.nih.gov, accessed on 6 November 2025) for identification, was followed by Clustal W analysis utilizing MegAlign software version 5.05 (DNASTAR Inc., Madison, Wisconsin, USA) for phylogenetic assessment (Thompson et al. 1994).

### Statistical analysis

All experiments were conducted following a randomized complete block design (RCB). Each treatment included at least ten biological replicates and three technical replicates, with results expressed as mean ± standard deviation (SD). Statistical analyses were conducted using one-way analysis of variance (ANOVA) in CoStat software (Version 6.3). Mean comparisons among treatments were carried out using Duncan’s multiple range test (DMRT) at a significance level of P ≤ 0.05.

## Results

### Isolation and purification of NBF

**Fig. 1:**
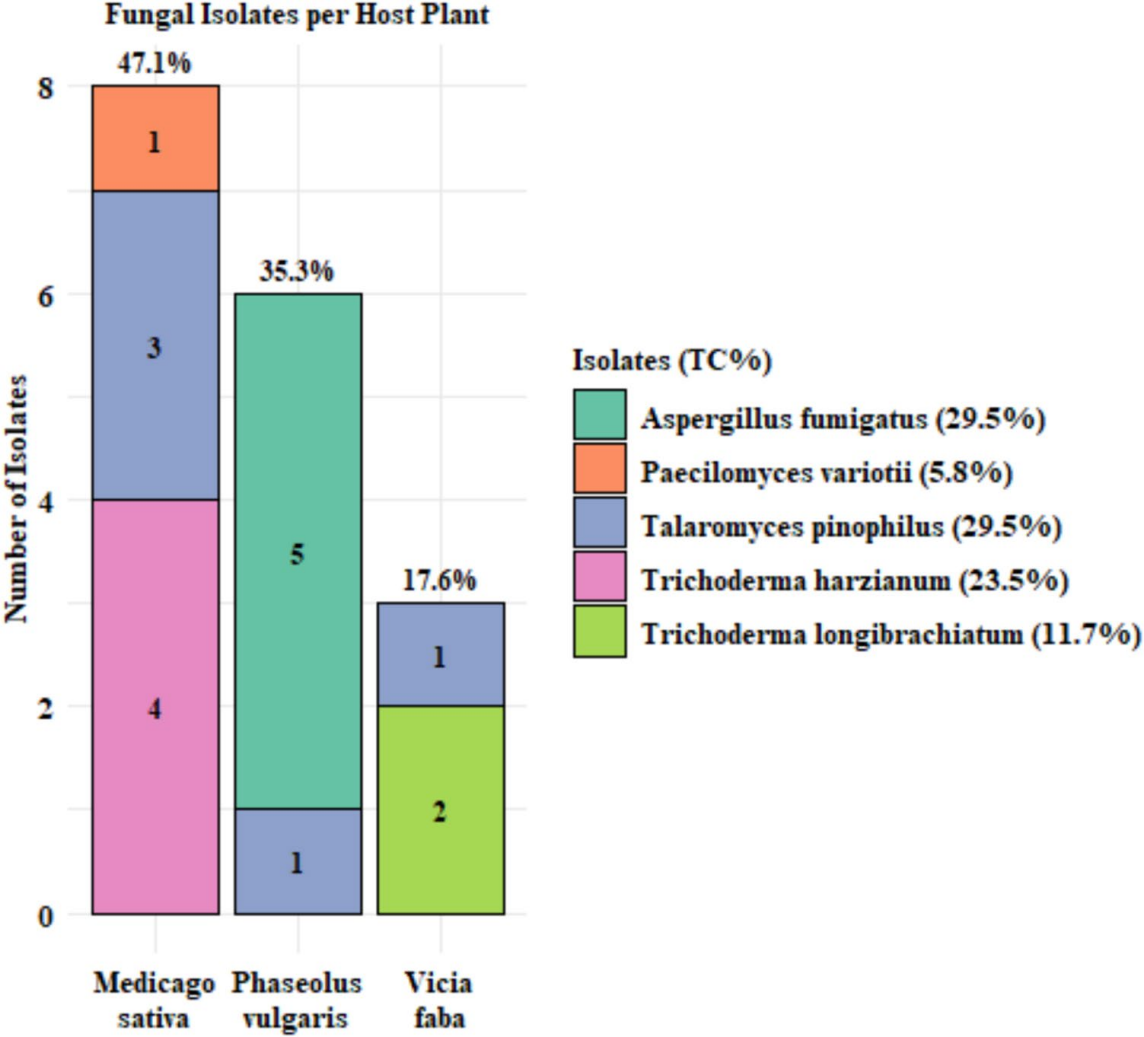
Distribution of the 17 nodule-borne fungal isolates (NBF) recovered from three host plants. Numbers inside each bar represent the total number of isolates obtained per host, whereas the percentages above the bars indicate the proportional contribution of each plant species to the overall isolate pool. The legend identifies each fungal species along with its total contribution (TC%) across all hosts

Seventeen plant-associated nodule-borne fungi (NBF) were isolated from the nodules of *Medicago sativa*, *Phaseolus vulgaris*, and *Vicia faba*. *M. sativa* yielded the highest number of isolates (8; 47.1%), followed by *P. vulgaris* (6; 35.3%), while *V. faba* had the lowest (3; 17.6%). The isolates belonged to five fungal species: *Aspergillus fumigatus* (29.5%), *Paecilomyces variotii* (5.8%), *Talaromyces pinophilus* (29.5%), *Trichoderma harzianum* (23.5%), and *Trichoderma longibrachiatum* (11.7%). *Talaromyces pinophilus* was detected in all three host plants, whereas other species showed more host specificity. The stacked bar chart **(**Fig. 1**)** illustrates the number of isolates per host plant, with the relative contribution of each species (TC%) represented by the fill gradient of each bar. This highlights that *A. fumigatus* and *T. pinophilus* dominate the fungal community across the examined hosts, suggesting potential for targeted biocontrol applications. Multiple fungal taxa were successfully isolated from legume nodules (Fig. 1); however, their ecological role within the nodules remains to be fully clarified.

Across all host species, several fungal taxa-particularly *Talaromyces pinophilus* and *Trichoderma* spp. were consistently recovered from multiple nodules collected from different plants and biological replicates. These repeated recoveries support the view that at least some isolates represent stable nodule endophytes rather than accidental contaminants or transient colonizers.

### Morphological identification of NBF

**Fig. 2:**
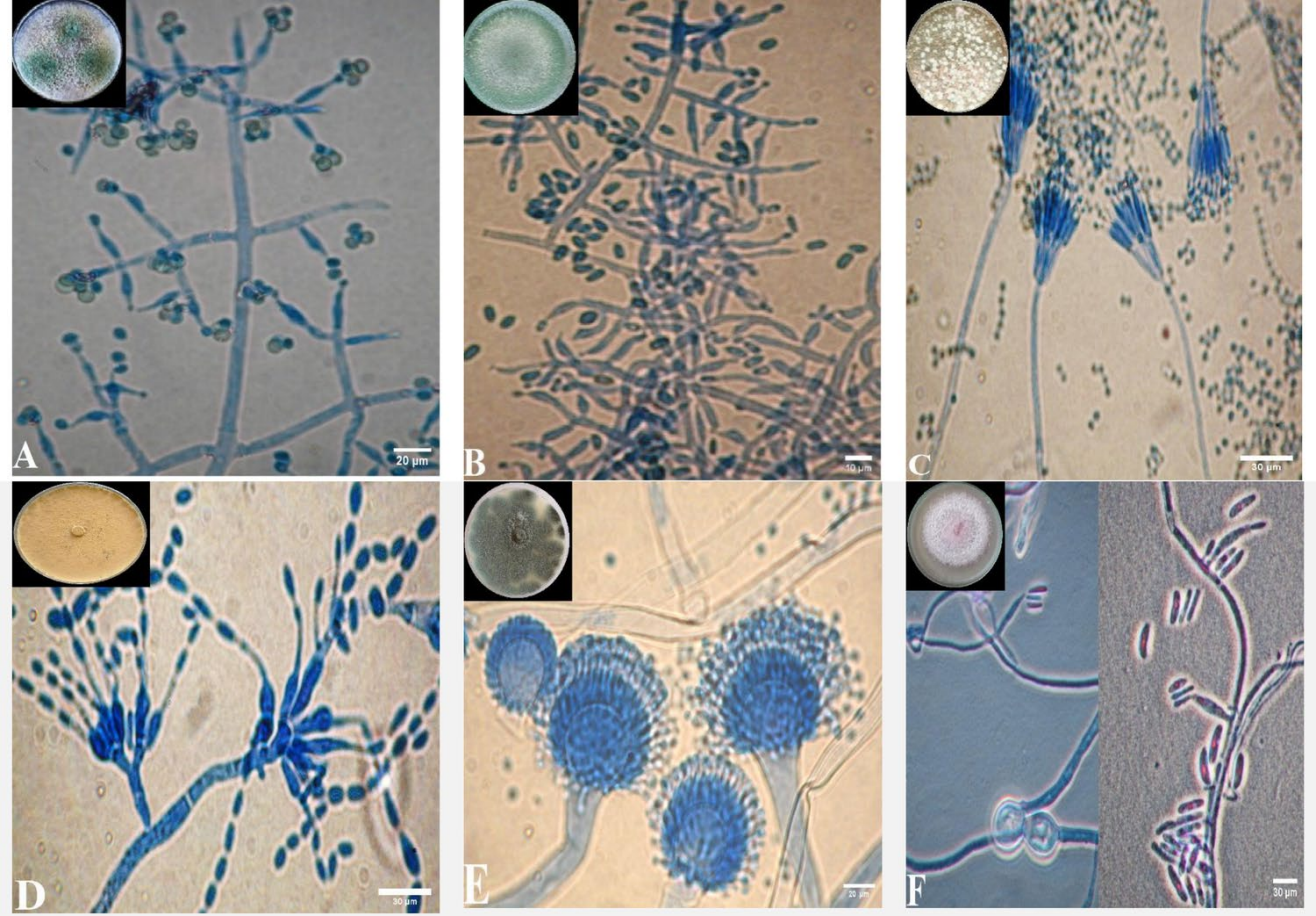
Colony morphology on PDA at 28 °C after 8 days and microscopic view showing conidiophores producing long chains of conidia. **A**: *Trichoderma harzianum*_20µm, **B**:* Trichoderma longibrachiatum*_10µm, **C**:* Talaromyces pinophilus_*30µm, **D**:* Paecilomyces variotii _*20µm, **E**: *Aspergillus fumigatus_*20µm, **F**: *Fusarium oxysporum* f. sp*. phaseoli_*30µm

Morphological characteristics of NBF isolates and fungal pathogen (Fop) isolated from wilted *P. vulgaris* were summarized as shown in **(**Fig. 2**)**. Only seven isolates were identified as follows: *Trichoderma harzianum* **(**Fig. 2 A**)** forms fast-growing colonies with a cottony or floccose texture, often appearing green due to abundant conidiation. Conidiophores are highly branched, with phialides producing smooth, globose to subglobose conidia. *Trichoderma longibrachiatum* (Fig. 2 B) exhibits similar morphological traits but is distinguished by its longer conidiophores and ellipsoidal to cylindrical conidia. *Talaromyces pinophilus* (Fig. 2 C) is characterized by its fast-growing colonies that produce dense, velvety, and often zonated textures, typically exhibiting yellow to orange hues. Conidiophores are biverticillate, bearing phialides that produce smooth-walled, spherical to subspherical conidia. *Paecilomyces variotii* (Fig. 2 D) produces colonies with a powdery to velvety texture, typically in shades of yellow–brown to olive. Its conidiophores are irregularly branched, bearing lanceolate phialides that produce smooth, ellipsoidal to fusiform conidia, often forming long chains. *Aspergillus fumigatus* (Fig. 2E) is characterized by its fast-growing, velvety colonies that appear blue-green to gray, with conidiophores bearing phialides that produce chains of spherical to subglobose conidia. Moreover, conidiophores are typically smooth-walled and columnar, with a uniseriate or biseriate arrangement of phialides. *F*.* oxysporum* f. sp*. phaseoli* (Fig. 2 F), a plant pathogen, produces cottony colonies with a pale pink to purple hue. It forms curved macroconidia with three to five septa, along with smaller oval to kidney-shaped microconidia, and often produces chlamydospores in older cultures.

### Assessing NBF isolates for plant growth‑promoting traits

PGP traits for the selected nodule-associated fungal endophytes, upon these tests, are described in Table 1. Quantitatively, *T. harzianum* exhibited the highest IAA production (54.04 μg/mL), followed by *P. variotii *(47.38 μg/mL) and *Aspergillus fumigatus* (44.66 μg/mL). In contrast, GA_3_ production was relatively uniform across isolates, with *T. pinophilus* (TP-BAC2) showing the highest value (49.06 μg/mL). For ammonium (NH_4_) production,, *T. harzianum* again led (19.05 mg/mL), while *T. pinophilus* (TP-BAC3) had the lowest (1.25 mg/mL). Phosphate solubilization was notably high in *T. pinophilus* (TP-BAC2) (534.64 mg/mL), whereas *P. variotii* and *Aspergillus fumigatus* showed the lowest values (102.29 and 167.58 mg/mL, respectively).

**Table 1:**
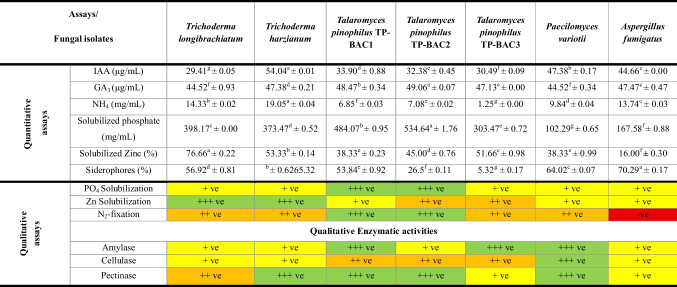
Criteria for plant growth promotion and enzyme activity of nodule-associated fungal endophytes isolated from *Medicago sativa*, *Phaseolus vulgaris*, and *Vicia faba*

Value: mean ± standard deviation (SD). Means denoted by the same letter(s) are not statistically different, according to the Duncan test (P < 0.05). Results of N₂ fixation derived from preliminary evidence. The positive results expressed by green color for , orange for , yellow for , and red for negative results

Zinc was most significantly enabled by *T. longibrachiatum* (76.66%), while *A. fumigatus* performance was poor (16.00%). Siderophore production was the most pronounced by *A. fumigatus* (70.29%) and *T. harzianum* (65.32%), however, *T. pinophilus* (TP-BAC3) showed the minimal productivity (5.32%). Qualitatively, the isolates showed positive phosphate and zinc solubilization, with *T. pinophilus* (TP-BAC1 and TP-BAC2) exhibiting stronger phosphate solubilizing activity compared to others. The isolates demonstrated growth on nitrogen-free medium, suggesting a potential nitrogen-fixing capability. Nitrogen fixation was positive for all isolates except *A. fumigatus*. While fungal isolates grew on nitrogen-free medium, indicating the ability to assimilate nitrogen under limited conditions, this does not constitute direct evidence of biological N₂ fixation.

**Fig. 3:**
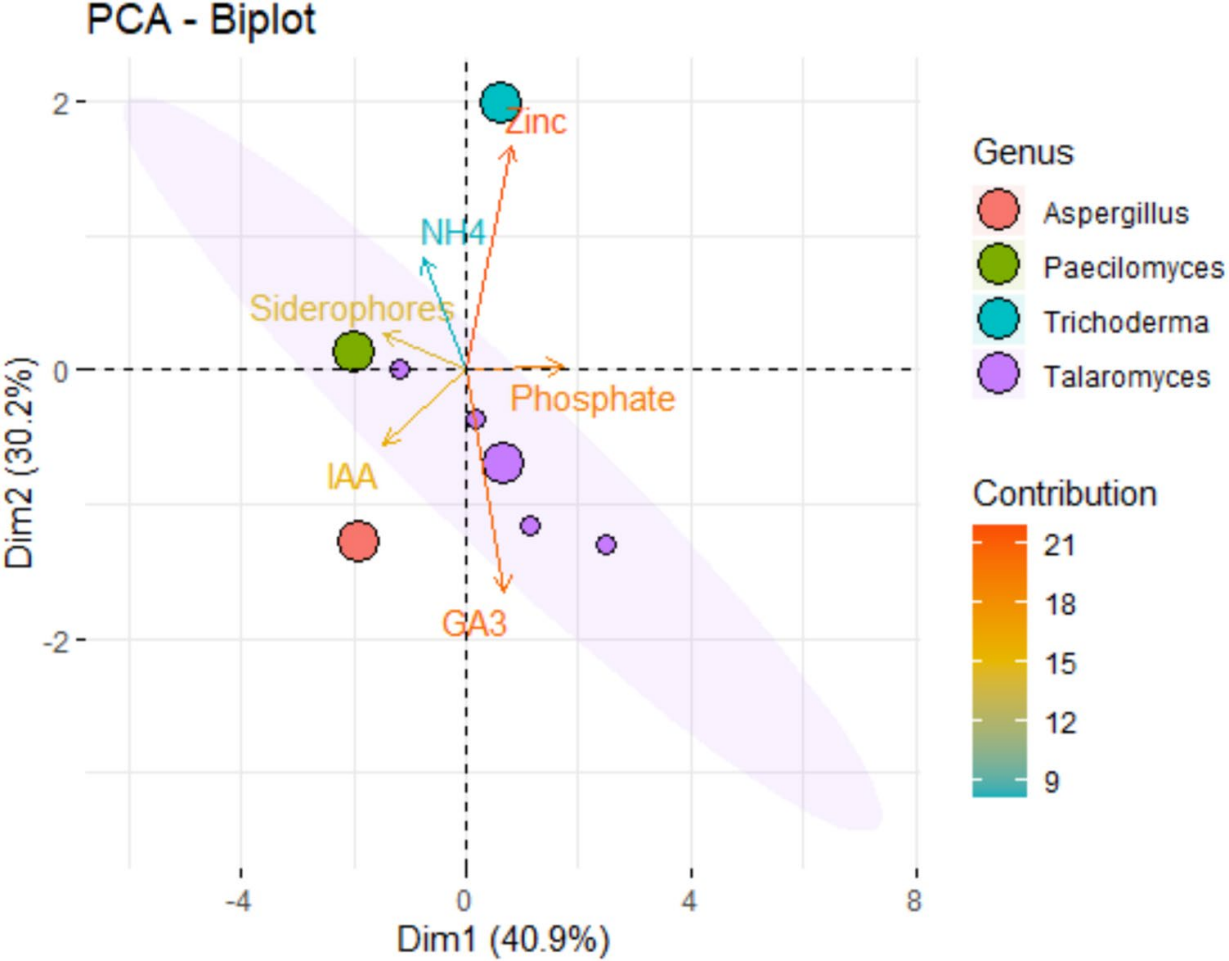
Principal component analysis (PCA- biplot) of the assessed plant growth-promoting traits for fungal isolates, explaining together 71.1% of the total variance. Arrows represent the contribution and direction of each variable, while points indicate individual fungal strains labeled by name. Ellipses denote the 95% confidence intervals for each genus group

Qualitatively, *T. pinophilus* isolates and *P. variotii* exhibited potential amylase and cellulase activities, whereas the pectinase activity was uniformly high across most isolates, except for *Aspergillus fumigatus*, which showed a moderate activity (+ ve). The PCA biplot analysis illustrates the distribution of four fungal genera: *Aspergillus*, *Paecilomyces*, *Trichoderma*, and *Talaromyces* based on the aforementioned results (Fig. 3). *Aspergillus* (orange circles) aligned closely with IAA production. *Paecilomyces* (dark green circles) is associated with NH_₄_ production and siderophores, while *Talaromyces* (violet circles) showed a relationship with GA_₃_ production and Phosphate solubilization. *Trichoderma* (mint green circles) appears broadly distributed and shows a strong Zinc solubilization, indicating diverse metabolic traits. *Trichoderma* showed a broad metabolic potential, while *Paecilomyces* may be more linked to ammonium metabolism.

### Antagonistic activity of fungal crude filtrates

**Fig. 4:**
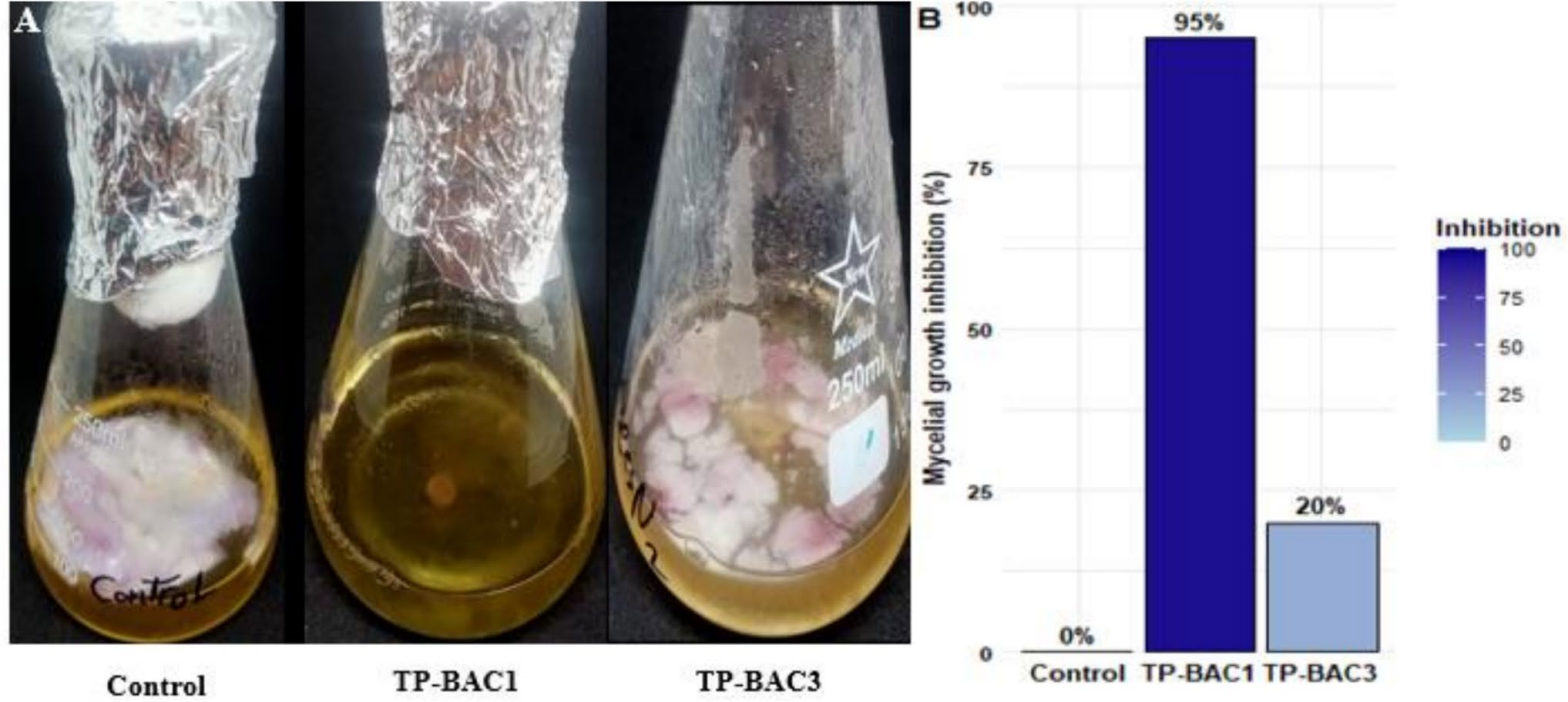
The antagonistic activity of crude filtrates of nodule-borne fungi (NBF) against *Fusarium oxysporum* f. sp. *phaseoli*. (**A**) Visual comparison of fungal growth inhibition. (**B**) Mycelial growth inhibition (%) showing the highest effect in *Talaromyces pinophilus* TP-BAC1compared to control and *Talaromyces pinophilus* TP-BAC3 as lowest effect

The findings demonstrated that, in the control scenario, *F. oxysporum* f. sp. *phaseoli* (Fop) exhibited autonomous growth; however, in the presence of specific NBF crude filtrates, including *Trichoderma longibrachiatum*, *Trichoderma harzianum*, *Talaromyces pinophilus* (TP-BAC1), *Talaromyces pinophilus* (TP-BAC2), *Paecilomyces variotii*, and *Aspergillus fumigatus*, there was a notable inhibitory effect on Fop's growth. Conversely, *Talaromyces pinophilus* (TP-BAC3) did not inhibit the growth of Fop compared to the other isolates, indicating an absence of inhibitory compounds or mechanisms under the experimental conditions. Figure 4 illustrates considerable disparities in antagonistic activity between the most potent (TP-BAC1) and the least (TP-BAC3) effective isolates compared to the control.

### Antifungal activity of ethyl acetate (EtOAc) extract of crude filtrates

**Fig. 5:**
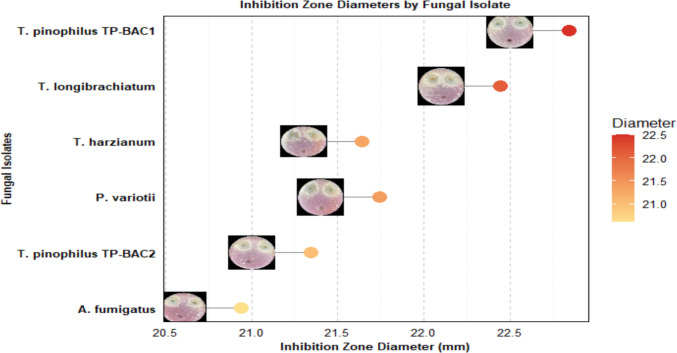
Diameters of inhibition zones of nodule-associated fungal endophytes against* F. oxysporum* f. sp. *phaseoli*. Images of the colony are displayed on the left, with colored spots representing diameters on the right. Horizontal lines link images to points; the color gradient represents diameter values

Figure 5 demonstrates the antifungal activity of the nodule-borne fungal isolates (NBF) against Fop, as indicated by the diameter of the inhibition zone (mm) formed around the wells. Overall, the data provide insights into the efficacy of the antifungal treatment of different fungal isolates against Fop, with varying levels of inhibition observed. The results indicated the varying levels of inhibitory effects, with the largest inhibition zone observed at 22.5 mm, followed by 22 mm for *Talaromyces pinophilus* TP-BAC1, *Trichoderma longibrachiatum,* and 21 mm for each *P. varioti*i,* Trichoderma harzianu*m, and *Talaromyces pinophilus* TP-BAC2. According to the plot, the smallest inhibition zone recorded was 20.6 mm for *Aspergillus fumigatu*s.

### Secondary metabolites of NBF

**Fig. 6:**
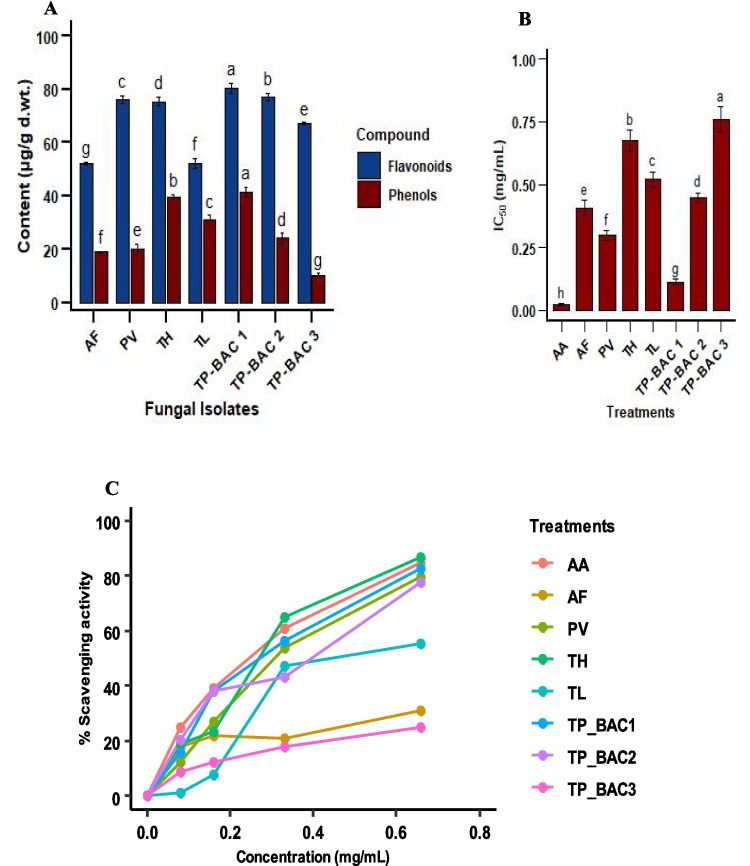
Biochemical profile of NBF: (**A**) total phenolic and flavonoid contents, (**B**) IC₅₀ values, and (**C**) percentage scavenging capability of NBF, underscoring its potential in bioactive synthesis. Abbreviations: AA, Ascorbic acid; AF, *Aspergillus fumigatus*; PV, *Paecilomyces variotii*; TH, *Trichoderma harzianum*; TL, *Trichoderma longibrachiatum*; TP-BAC1, *Talaromyces pinophilus* (TP-BAC1); TP-BAC2, *Talaromyces pinophilus* (TP-BAC2); TP-BAC3, *Talaromyces pinophilus* (TP-BAC3)

Flavonoid and phenolic contents of the ethyl acetate fractions of the nodule-borne fungal isolates were disparate and showed vast intraspecific diversity within the species. The highest flavonoid content was detected in *Talaromyces pinophilus* TP-BAC1 (89.34 µg/mL), which differed significantly from all others, while *Talaromyces pinophilus* TP-BAC2 (88.14 µg/mL), in regarding with *Trichoderma harzianum* (79.21 µg/mL) and *Paecilomyces variotii* (80.01 µg/mL), flavonoid concentrations were statistically similar but lower than *Talaromyces pinophilus* TP-BAC1 and *Talaromyces pinophilus* TP-BAC2. On the other hand, *Trichoderma longibrachiatum* (52.18 µg/mL) and *Aspergillus fumigatus* (52.11 µg/mL) had the lowest flavonoid content. Phenol contents were similar, with the majority being produced by *Talaromyces pinophilus* TP-BAC1 (41.33 µg/mL) and *Trichoderma harzianum* (39.35 µg/mL), while *Talaromyces pinophilus* TP-BAC3 (10.31 µg/mL) produced the least amount. Interestingly, *Talaromyces pinophilus* TP-BAC2 gave high flavonoid content but extremely low content of phenols (24.1 µg/mL), reflective of preferential metabolic diversion in the direction of flavonoid biosynthesis. *Paecilomyces variotii* gave intermediate flavonoid content (20 µg/mL) but low phenol content (Fig. 6 A).

The IC50 values of fungal extracts (ethyl acetate) for DPPH radical scavenging activity exhibited considerable variability. The isolates with IC50 values between 0.1 and 0.3 mg/mL demonstrated robust antioxidant activity, indicating their potential as effective free radical scavengers. Isolates exhibiting IC50 values within the range of 0.4—0.6 mg/mL demonstrated moderate activity, but those with values over 0.7 mg/mL displayed comparatively diminished antioxidant capability. These findings highlight the potential of certain fungal isolates, particularly those with lower IC50 values, as sources of natural antioxidants. Among the tested isolates, *Talaromyces pinophilus* TP-BAC1 exhibited the lowest IC_50_ (0.113 mg/mL), indicating the highest potency, followed by *Paecilomyces variotii* (0.297 mg/mL) and *Trichoderma longibrachiatum* (0.375 mg/mL) (Fig. 6 B). In the scavenging assay, all isolates displayed concentration-dependent activity, with *Talaromyces pinophilus* TP-BAC1 and *Trichoderma harzianum* achieving notable scavenging rates of 86.66% and 82.57%, respectively, at 0.66 mg/mL (Fig. 6 C). The observed antioxidant effects may result from both fungal metabolite contributions and priming of host defense pathways, as suggested by our results.

### Fungal isolates' effect on *P. vulgaris resistance to* Fop infection

**Fig. 7:**
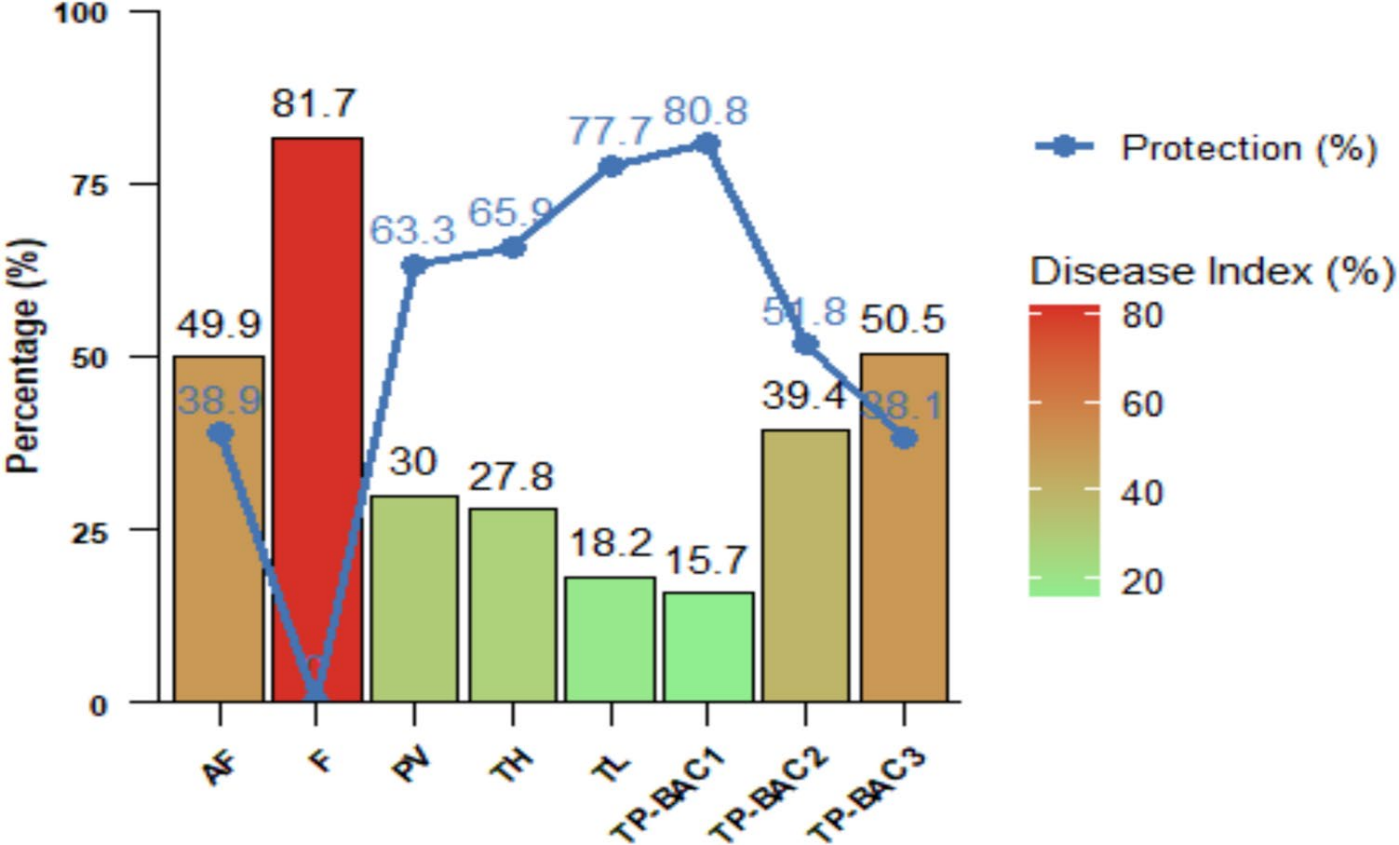
Disease Severity (%) and Protection (%) across different NBF treatments on 45 day- old *P. vulgaris* plants. Bars represent DS with a color gradient from green (low severity) to red (high severity). The blue line and points indicate the Protection percentage for each treatment. Values above bars and points show exact percentages. Abbreviations: AF, *Aspergillus fumigatus*; F, *Fusarium oxysporum*; PV, *Paecilomyces variotii*; TH, *Trichoderma harzianum;* TL, *Trichoderma longibrachiatum;* TP-BAC1, *Talaromyces pinophilus* (TP-BAC1); TP-BAC2, *Talaromyces pinophilus* (TP-BAC2); TP-BAC3, *Talaromyces pinophilus* (TP-BAC3)

Disease severity (DS) and protection levels in *P. vulgaris* plants infected with Fop were significantly influenced by the applied fungal treatments, as illustrated in Fig. 7. The infected control exhibited the highest DS (81.7%). In contrast, plants treated with *Talaromyces pinophilus* TP-BAC1 showed the lowest DS (15.72%) and consequently the highest protection percentage (80.75%), indicating its strong biocontrol potential. Treatments with *Trichoderma longibrachiatum*, *Trichoderma harzianum*, *Paecilomyces variotii,* and *Talaromyces pinophilus* TP-BAC2 also provided significant disease reduction and moderate protection, though less effective than* Talaromyces pinophilus* TP-BAC1. Meanwhile, isolates such as *Aspergillus fumigatus* and *Talaromyces pinophilus* TP-BAC3 exhibited relatively higher disease indices and lower protection.

**Fig. 8:**
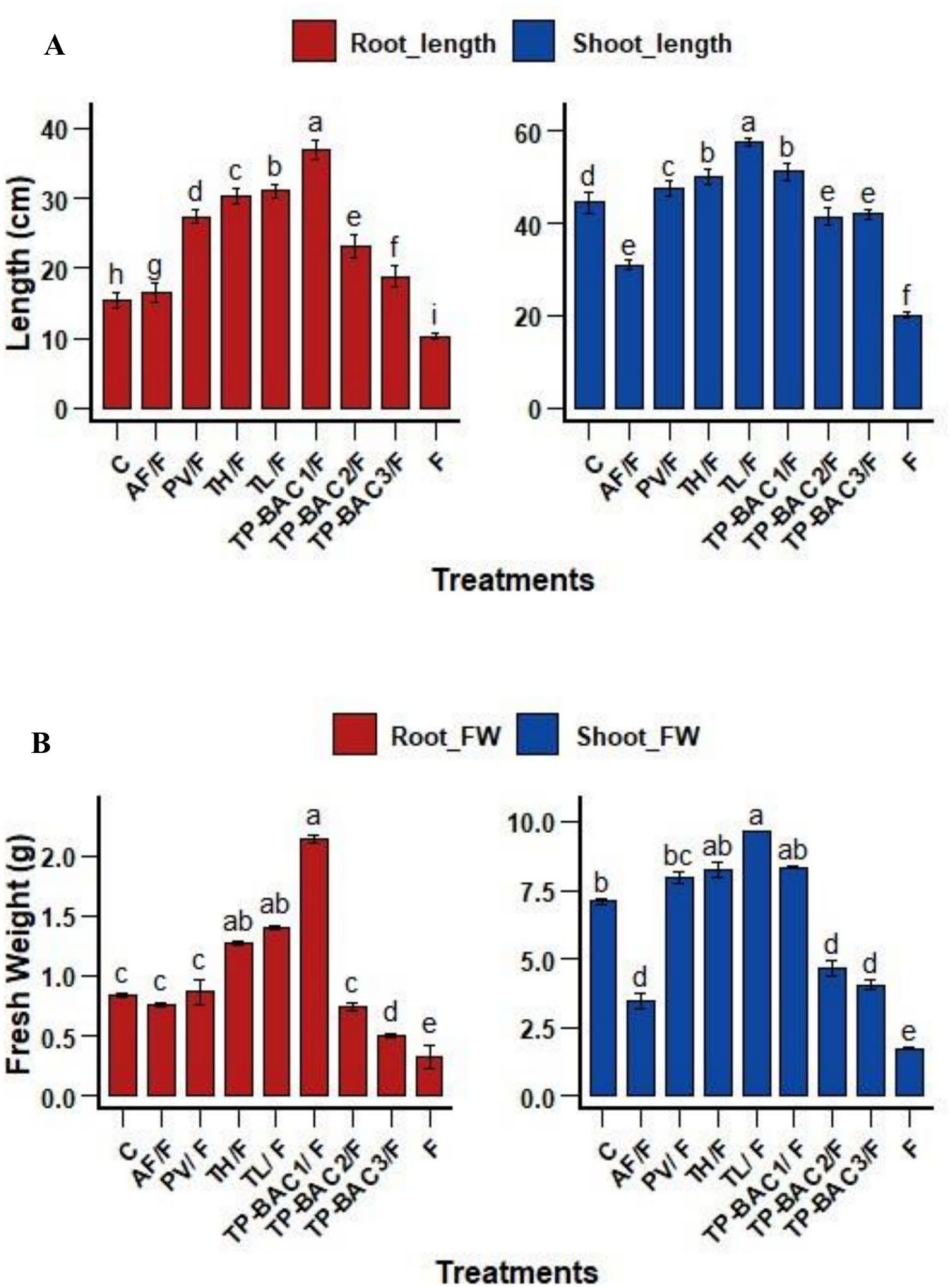
Impact of fungal treatments on the growth metrics: **A**) shoot and root length, **B**) shoot and root fresh, **C**) dry weight, and **D**) leaf count of *P. vulgaris* after 45 days. Abbreviations: C = Control, TP-BAC1/F = *T. pinophilus* TP-BAC1/F, TL/F = *T. longibrachiatum*/F, TH/F = *T. harzianum*/F, PV/F = *P. variotii*/F, TP-BAC2/F = *T. pinophilus* TP-BAC2/F, AF/F = *A. fumigatus*/F, TP-BAC3/F = *T. pinophilus* TP-BAC3/F, where F represents *F. oxysporum* only. The Duncan's test indicated that distinct superscript letters signify significant differences between each bar (P ≤ 0.05)

The *P. vulgaris* plants infected with *F. oxysporum* f. sp. *phaseoli* exhibited a notable reduction in shoot length (20.17%), root length (10.21%), and leaf count (2.66%) compared to the healthy control plants, as illustrated in Fig. 8. The findings demonstrated that multiple growth metrics (shoot length, root length, fresh and dried weight of shoots and roots, and leaf count) were greatly enhanced by the application of NBF isolates. Conversely, infected plants treated with the evaluated fungal isolates (*Talaromyces pinophilus* TP-BAC1, *Trichoderma longibrachiatum*, and *Trichoderma harzianum*) showed promising recovery, with *Talaromyces pinophilus* TP-BAC1 being the most effective isolate.

**Fig. 9:**
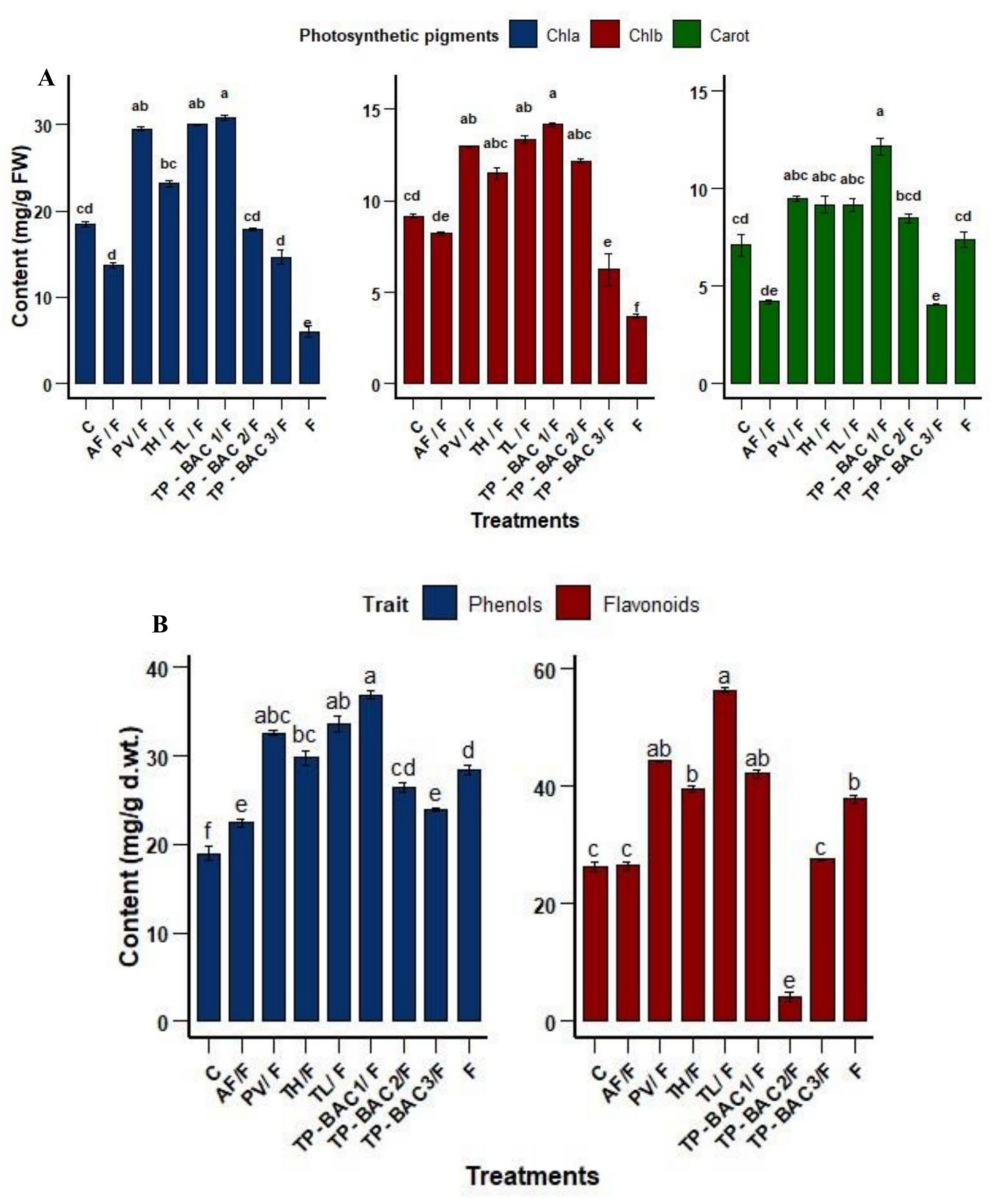
Impact of fungal treatments on **A**) photosynthetic pigments (Chlorophyll a, Chlorophyll b, Carotenoids), **B**) total phenolic and flavonoid concentrations, **C**) malondialdehyde and hydrogen peroxide levels, **D**) membrane damage and stability index of *P. vulgaris* after 45 days. Abbreviations: (C = Control, AF/F = *A. fumigatus*/F, PV/F = *P. variotii*/F, TH/F = *T. harzianum*/F, TL/F = *T. longibrachiatum*/F, TP-BAC1/F = *T. pinophilus* TP-BAC1/F, TP-BAC2/F = *T. pinophilus* TP-BAC2/F, TP-BAC3/F = *T. pinophilus* TP-BAC3/F, where F represents *F. oxysporum* f. sp. *phaseoli* only. The Duncan's test indicated that distinct letters signify significant differences among each bar (P ≤ 0.05)

The results demonstrated the influence of *F. oxysporum* infection and the protective roles of different fungal endophytes on the photosynthetic pigments (chlorophyll a, chlorophyll b, and carotenoids) in *P. vulgaris* (Fig. 9 A). The infected control had a severe reduction in pigment concentrations, recording the lowest levels of chlorophyll a (6.02 mg/g f.wt.), chlorophyll b (3.7 mg/g f.wt.), and carotenoids (7.4 mg/g f.wt.), signifying considerable photosynthetic dysfunction resulting from pathogen stress. Conversely, the healthy control exhibited moderate pigment levels, indicating a baseline physiological condition. Treatment with *Talaromyces pinophilus* TP-BAC1 yielded the greatest amounts of chlorophyll a (30.79 mg/g f.wt.), chlorophyll b (14.18 mg/g f.wt.), and carotenoids (12.17 mg/g f.wt.), above those of the healthy control, indicating a significant protective or stimulatory effect. Additional treatments, specifically *Trichoderma longibrachiatum* and *Pacelomyces varrotii*, markedly reinstated pigment content to levels comparable to or surpassing those of healthy controls. In contrast, treatments with *Aspergillus fumigatus,* and *Talaromyces pinophilus* TP-BAC3 exhibited diminished efficacy compared to the infected control.

Plant secondary metabolites, phenolic and flavonoid, contents varied notably among treatments, with clear differences between infected, healthy, and fungal-treated plants (Fig. 9 B). The healthy control showed baseline levels of phenols (18.98 µg/g) and flavonoids (26.3 µg/g), while *F. oxysporum* infection resulted in a marked, substantial increase in both phenols (28.34 µg/g) and flavonoids (37.87 µg/g), reflecting a typical plant defense response to pathogenic stress. Several fungal treatments, particularly *Trichoderma longibrachiatum* (33.65 and 56.34 µg/g for phenols and flavonoids, respectively), *Talaromyces pinophilus* TP-BAC1 (36.9 and 42.12 µg/g), and *Pacelomyces varrotii* (32.56 and 44.32 µg/g), resulted in even higher levels than Fop-treated plants, suggesting their assumed role in priming or enhancing the plant's antioxidant resistance mechanisms. Conversely, *Aspergillus fumigatus* and *Talaromyces pinophilus* TP-BAC3 treatments exhibited relatively low phenol and flavonoid contents, in some cases near or below infection levels, implying weaker bioactivity or limited capacity to induce chemical defense responses.

Data from (Fig. 9 C & D) present key physiological and stress-related parameters in plants treated with different fungal isolates, revealing significant variations in membrane integrity, stress tolerance, and oxidative status. *Talaromyces pinophilus* TP-BAC1 and *Trichoderma harzianum* demonstrated the most favorable outcomes in mitigating oxidative stress. Both isolates maintained low levels of malondialdehyde (MDA: 0.12 and 0.15 µmol/g f. wt., respectively), indicating reduced lipid peroxidation, and exhibited the lowest hydrogen peroxide (H₂O₂) content (0.64 and 0.85 µmol/g f. wt.), reflecting enhanced cellular protection. Furthermore, these treatments showed the lowest membrane injury index (MII: 23.52 and 32.03 cm) and the highest membrane stability index (MSI: 71.48 and 67.97 cm), confirming their effectiveness in preserving membrane integrity under stress conditions. In contrast, *Fusarium oxysporum* exhibited severe stress induction, with the highest MII (89.00 cm), lowest MSI (11.00 cm), alongside elevated MDA (0.58 µmol/g f. wt.) and H₂O₂ (1.9 µmol/g f. wt.), reflecting significant membrane damage and oxidative stress, followed by *Aspergillus fumigatus* and *Talaromyces pinophilus* TP-BAC3 strain, which raised up the stress responses, with MII values ranging from 60.50 cm to 52.83 cm and moderate oxidative markers. Notably, *Talaromyces pinophilus* TP-BAC3 displayed strong membrane stability (MSI: 71.48 cm), suggesting strain-specific mechanisms of stress adaptation.

### Multivariate analysis: Two-way clustering dendrogram with heatmap

The clustering and heatmap analysis revealed distinct functional groups of isolates, which corresponded with differences in biocontrol performance and growth-promotion activity, highlighting the importance of strain-specific selection for practical applications. Moreover, this two-way hierarchical clustering dendrogram presented in the heatmap provides a comprehensive multivariate analysis of the relationships between treatments and traits. This method groups both treatments (rows) and traits (columns) based on the similarity of their Z-score profiles, allowing for the identification of patterns and associations across the dataset. For trait clustering (columns), the dendrogram at the top of the heatmap clusters traits that exhibit similar expression patterns across treatments. Traits like MII, H₂O₂, and MDA may form a distinct cluster, suggesting they induce similar biochemical or physiological responses. In contrast, treatments such as LN, ShD, ShF, and ShL may cluster separately, reflecting a different mode of action or lower overall activity. Also, RL, RD, Chla, Chl b, and MSI traits are grouped in one cluster. Similarly, TPC, TFC, RF, and Carot traits were found in the other subcluster.

**Fig. 10:**
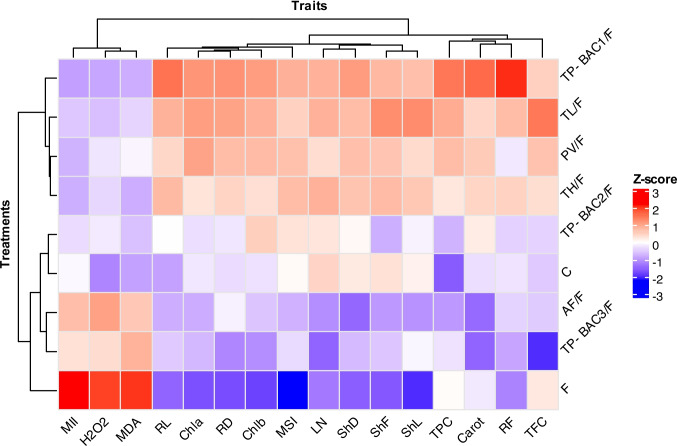
Two-way hierarchical clustering with heatmap of standardized trait values (Z-scores) across different fungal treatments and controls. Rows represent treatments, and columns represent morphological, physiological, and biochemical traits. Color intensity indicates the relative increase (red) or decrease (blue) of each trait after standardization. Abbreviations: ShL; Shoot length, ShF; Shoot fresh weight, ShD; Shoot dry weight, RL; Root Length, RF; Root fresh weight, RD; Root dry weight, LN; Leaf number, Chl a; Chlorophyll a, Chl b; Chlorophyll b, Carot; Carotenoids, TPC; Total phenol content, TFC; Total flavonoid content, MII; Membrane injury index, MSI; Membrane stability index, H_2_O_2_; Hydrogen Peroxide, and MDA; Malondialdehyde

Concerning treatment clustering (rows), the dendrogram on the left groups treatments based on their overall trait expression profiles. Treatments can be divided into two main clusters; one cluster was subdivided into two subclusters. In cluster I, F treatment is separated in alone cluster, whereas the other subcluster had TP-BAG3/F, AF/F, C, and TP-BAG2/F. Cluster II is also divided into two subclusters, including TP-BAG1/F, TL/F, TH/F, and PV/F, which may cluster together, indicating they respond similarly to various treatments and may be functionally related or co-regulated. The heatmap’s color gradient-from blue (low Z-scores) to red (high Z-scores)-visually represents the relative intensity of each trait under each treatment. Clusters with predominantly red hues indicate treatments that strongly upregulate certain traits, while blue clusters suggest suppression or low expression. The heatmap analysis revealed distinct patterns of trait expression across the evaluated treatments, based on Z-score normalization (Fig. 10).

Traits such as MII, H₂O₂, and MDA exhibited strong positive associations with treatment like F treatment, indicated by intense red coloration, suggesting elevated activity or accumulation of these traits under these conditions. Conversely, traits including root and shoot growth parameters, chlorophyll content, and membrane stability showed predominantly negative Z-scores (blue), reflecting reduced trait expression under F treatment. Intermediate responses were observed in the F treatment, such as TPC, TFC, and Carot, which displayed mixed profiles across traits. On the other hand, TP-BAG1/F treatment showed mostly positive intercorrelation with all traits except MII, H_2_O_2,_ and MDA. These results highlight the differential impact of treatments on biochemical and physiological traits, suggesting potential pathways for targeted enhancement or suppression in future applications.

Out of seven fungal isolates tested for their biocontrol potential against a single aggressive pathogenic isolate, *Fusarium oxysporum* f. sp. *Phaseoli* (FOP), one isolate (*Talaromyces pinophilus* TP-BAC1) exhibiting the highest biocontrol activity, and the pathogenic isolate itself were selected for molecular identification. The isolates were molecularly identified by ITS region of rRNA sequence analysis. The results showed that the strain of *Talaromyces pinophilus* TP-BAC1 showed 99.20%—100% identity and 100% coverage with several strains of *T. pinophilus,* including the type material CBS 631.66_ NR_111691. Likewise, the strain of FOP showed 100% identity and 100% coverage with several strains of *F. oxysporum.* After being submitted to the GenBank repository at https://www.ncbi.nlm.nih.gov/nuccore/, the obtained sequences were assigned the accession numbers PX496006 and PX496026 in GeneBank for TP-BAC1 and FOP, respectively.

## Discussion

Plant defense against pathogens relies on a complex immune system that enables early recognition of infection and activation of protective responses. This immunity involves structural barriers, production of defense-related metabolites, and induction of antioxidant and signaling pathways that collectively restrict pathogen development. Such mechanisms are closely linked to plant resistance, and beneficial microbes, such as plant growth-promoting fungi (PGPF), can enhance these natural defense responses and reduce disease severity (Jones and Dangl 2006).

Root nodules, traditionally known for harboring nitrogen-fixing rhizobia, also serve as ecological niches for diverse fungal endophytes with beneficial traits (Hassen et al. 2025). Recent evidence showed that endophytic fungi associated with legumes exhibit diverse traits associated with plant growth promotion, including phosphate solubilization, IAA production, siderophore secretion, and antioxidant activity, which contribute to nutrient acquisition and systemic resistance against pathogens (Verma et al. 2021). In this study, seventeen filamentous fungal isolates were obtained from the nodules of *Medicago sativa*, *Phaseolus vulgaris*, and *Vicia faba,* and these were *Aspergillus fumigatus*, *Paecilomyces variotii*, *Talaromyces pinophilus*, *Trichoderma longibrachiatum*, and *T. harzianum* (Fig. 1). To our knowledge, this is the first report of these fungal taxa inhabiting root nodules of these hosts, thereby broadening current understanding of the nodule-associated mycobiota, which has been less explored compared to rhizospheric or general endophytic fungi (Abd-Alla et al. 2024).

In this study, the recurrent detection of *Talaromyces pinophilus* and *Trichoderma* spp. across nodules of different host plants provides evidence that these fungi are stable nodule endophytes rather than incidental occupants. Persistent colonization by non-rhizobial microorganisms has been documented in several legumes, where fungi may enter developing nodules through infection threads or intercellular spaces and subsequently establish long-term residency. Such colonization pathways offer a mechanistic explanation for the stable presence of these taxa in our samples and highlight their potential functional relevance within the nodule microenvironment.

Nodule-associated fungi may act as symbionts, opportunists, or pathogens, with their interactions with rhizobia potentially shaping plant health and soil fertility. Several isolates from legume nodules have been shown to suppress *F. oxysporum* f. sp. *phaseoli* through mycoparasitism, hydrolytic enzymes, and competition (El-Sharkawy and Abdelrazik 2022). These fungi likely colonize nodules during ontogenesis via infection threads and benefit from the nutrient-rich environment (Martínez-Hidalgo and Hirsch 2017). The investigation revealed the highest fungal prevalence was observed in *M. sativa* at 47.1%, followed by *P. vulgaris* (35.3%) and *V. faba* (17.6%). The predominance of *Aspergillus fumigatus* and *Talaromyces pinophilus* (29.5% each) (Fig. 1) suggests ecological importance, though their absence in *P. vulgaris* and *V. faba* may indicate host specificity or microbial competition (Sánchez-López et al. 2018). Moreover, *Trichoderma harzianum* (23.5%) in *M. sativa* and *Trichoderma longibrachiatum* (11.7%) (Fig. 1) in *V. faba* highlight potential niche adaptation, while the reduced diversity in *V. faba* could reflect antimicrobial defenses or selective symbiosis (Mabood et al. 2014).

The fungal isolates displayed significant variation in plant growth-promoting (PGP) traits, highlighting their potential for agricultural applications. *Trichoderma harzianum* showed the highest IAA production, promoting root development (Li et al. 2025), while *Talaromyces pinophilus* (TP-BAC2) excelled in phosphate solubilization (Rana et al. 2025). High siderophore yield of *Aspergillus fumigatus* and *Trichoderma harzianum* supports their role in iron chelation (Sharma et al. 2023), whereas the high amylase and cellulase activities of *Talaromyces pinophilus* and *Paceliomyces variotii* suggest contributions to organic matter decomposition and soil fertility. The variability in nitrogen fixation and zinc solubilization emphasizes the need for strain-specific selection to optimize biofertilizer efficacy (Bhattacharjee et al. 2023). Collectively, these traits indicate that nodule-associated fungi could play a dual role as biofertilizers as well as biocontrol agents.

The antagonistic assays revealed that several nodule-borne fungi, including *Trichoderma longibrachiatum*, *Trichoderma harzianum*, *Talaromyces pinophilus* (TP-BAC1, TP-BAC2), *Pacelomyces variotii*, and *Aspergillus fumigatus*, significantly inhibited the growth of *F. oxysporum* f. sp. *phaseoli* (Fop), whereas *Talaromyces pinophilus* (TP-BAC3) showed no inhibitory effect relative to the other isolates (Figs. 4 & 5). The marked differences among the three *Talaromyces pinophilus* isolates highlight the well-recognized phenomenon of strain-level functional variability within filamentous fungi. Although these isolates belong to the same species, their contrasting biological activities likely reflect differences in secondary-metabolite biosynthetic capacity, regulation of hydrolytic enzymes, and genomic heterogeneity in pathways associated with antibiosis and plant–microbe interactions. Such intra-species divergence has been widely reported in fungal genera where metabolite production, enzyme activity, and stress-responsive traits can vary substantially between genetically distinct strains (Li et al., 2025).

This variability highlights strain-specific differences in antifungal capacity, likely mediated through hydrolytic enzymes, antibiotic production, VOCS, or competitive exclusion (Singh et al. 2024). Similar findings from nodules of *Glycine max* and *Cicer arietinum* further support the biocontrol potential of *Trichoderma* and *Talaromyces* species against *Fusarium* wilt (Goyal and Kalia 2020). Collectively, the dual role of these fungi in promoting plant growth and suppressing pathogens underscores their promise as eco-friendly bioinoculants for sustainable legume production. The highest inhibition (22.5 mm) (Fig. 5) coordinated earlier findings on *Trichoderma harzianum* and *Aspergillus flavus* (Al Mousa et al. 2021), while even modest zones (> 15 mm) remain biologically applicable (Hasan et al. 2023). Comparable mechanisms, including lipopeptide production by *F. solani* (Rakshit et al. 2025) and siderophore-mediated competition (Kajula et al. 2010), support the role of fungal endophytes as potential biocontrol agents, though field validation remains necessary (Hyde et al. 2024). Metabolite and antioxidant profiling revealed isolate-specific variation. *Talaromyces pinophilus* TP-BAC1 produced the highest phenolic (41.33 µg/mL) and flavonoid (89.34 µg/mL) contents, a result that was reported before, consistent with reports on *Talaromyces* spp. metabolic richness (Abbas et al. 2025). Similarly, *Trichoderma harzianum* displayed strong biosynthetic capacity (Singh et al. 2024), whereas *Trichoderma longibrachiatum* and *Aspergillus fumigatus* showed minimal productivity (Chen et al. 2023). These trends align with the known correlation between phenolic content and antioxidant potential (Sultana et al. 2021). DPPH assays confirmed high scavenging activity in *Trichoderma harzianum*, *Talaromyces pinophilus* TP-BAC1, *Paceliomyces variotii*, and *Talaromyces pinophilus* TP-BAC2 (77- 86%) (Fig. 6), comparable to other antioxidant-producing endophytes (Hashem et al. 2023). Overall, these findings highlight the dual role of nodule-associated fungi as biocontrol agents and metabolite producers, reinforcing their potential in sustainable agriculture.

Fungal diseases, particularly *Fusarium oxysporum*, are major threats to crop production due to their soil-borne nature and persistence in agricultural soils (Alamri et al. 2019). Biocontrol using PGPF offers a sustainable strategy by enhancing nutrient uptake, decomposing organic matter, and inducing systemic resistance (Abdelaziz et al. 2022). In this study, fungal strains including *Aspergillus fumigatus, Paceliomyces variotii*, *Talaromyces pinophilus* (TP-BAC1, TP-BAC2, and TP-BAC3), *Trichoderma longibrachiatum*, and *Trichoderma harzianum* were evaluated for biocontrol of Fusarium wilt in *Phaseolus vulgaris*. Among all, *Talaromyces pinophilus* TP-BAC1 exhibited the strongest growth-promoting effects, improving root length, biomass, and shoot development, consistent with reports on *Talaromyces* spp. as potent plant growth promoters, a result that was reported before Zhang et al. (2023). *Trichoderma longibrachiatum* and *Trichoderma harzianum* also enhanced plant performance, likely through systemic resistance induction and improved nutrient uptake (Kashyap et al. 2024), whereas *Aspergillus fumigatus* and *Fusarium oxysporum* impaired growth, reflecting their pathogenic potential, a prior observation (Poveda et al. 2024). The variability among *Talaromyces pinophilus* strains underscores the importance of selecting optimal isolates for agricultural application. Disease severity (PDI 81.7%) confirmed the destructive effect of the used isolate of *F. oxysporum* (Fig. 7), aligning with previous studies (Muhorakeye et al. 2024).

In this investigation, application of PGPF significantly reduced *Fusarium* wilt severity, with reductions ranging from 15.72% to 50.54%. Furthermore, the treatments conferred a high level of protection, with protection percentages varying between 38.13 and 80.75%, highlighting isolates' strong biocontrol potential. Based on the current findings, treatment with *Talaromyces pinophilus* TP-BAC1 isolate proved to be the most effective in reducing the percentage disease severity (PDS), offering the highest level of plant protection. This highlights the strong protective role of PGPF against Fusarium wilt, which documented the capacity of PGPF to inhibit the Fusarium pathogen (Attia et al. 2022). Infected control plants showed significant reductions in growth traits, which were expected to be due to *F. oxysporum* (Fig. 8 A, B, C & D) induced hormonal disturbances as reported previously, Hossain et al. (2017). In contrast, PGPF treatments markedly improved growth parameters, and this might be ascribed to the growth-promoting traits of the used isolates, such as the production of IAA, gibberellic acid, siderophores, and nutrient solubilization (Jaber and Alananbeh 2018). The obtained results are consistent with previous reports of fungal metabolites enhancing growth and defense (Yang et al. 2024). These results highlight the dual role of PGPF in promoting plant vigor and mitigating the pathogen effects.

Fusarial infection severely impaired photosynthesis, as evidenced by the substantial drop in chlorophyll a and b contents, while carotenoids increased, indicating oxidative stress and chloroplast damage (Kalra et al. 2020). Among the tested strains, *Talaromyces pinophilus* TP-BAC1 exhibited the strongest restorative effect on chlorophyll a and b, followed by *Trichoderma longibrachiatum*, *Paceliomyces variotii*, and *Trichoderma harzianum* whereas *Aspergillus fumigatus* and *Talaromyces pinophilus* TP-BAC3 were less effective (Fig. 9 A). Infected plants treated with *Talaromyces pinophilus* TP-BAC1, *Trichoderma longibrachiatum*, *Trichoderma harzianum*, *Paceliomyces variotii*, and *Talaromyces pinophilus* TP-BAC2 also showed marked enhancement in carotenoids, consistent with earlier findings on fungal endophytes improving photosynthetic pigments (Andrews et al. 2004). This enhancement may be linked to N₂ enrichment and improved nutrient assimilation (Musheer et al. 2021). Furthermore, PGPF isolates likely enhanced disease suppression by activating plant defense responces, leading to elevated pigment accumulation and induction of defense-associated metabolites including phytoalexins and pathogenesis-related (PR) proteins (Shoresh et al. 2010).

Pathogen invasion triggers the synthesis or accumulation of phenolic compounds, which restrict pathogen progression through their antimicrobial and antioxidative properties (Sharma et al. 2019). This study recorded the greatest phenolic content of infected plants with *Talaromyces pinophilus* TP-BAC1, followed by *Trichoderma longibrachiatum*, *Paceliomyces variotii*, and *Trichoderma harzianum* (Fig. 9 B). Phenolics are essential for regulating metabolism, promoting vegetative growth, synthesizing lignin, and facilitating defense responses (Tuladhar et al. 2021). Similarly, flavonoid contents significantly increased under Fusarium infection and were further enhanced by PGPF treatments, particularly *Trichoderma longibrachiatum*, *Paceliomyces variotii*, *Talaromyces pinophilus* TP-BAC1, and *Trichoderma harzianum* (Fig. 9 B). The accumulation of phenols and flavonoids is well documented in plant disease resistance, and contributed to antioxidant activity, free radical scavenging, and reinforcement of the cell wall, thereby enhancing tolerance to biotic stress (Li et al. 2022).

Although three isolates of *T. pinophilus* were recovered, only TP-BAC1 exhibited strong antagonistic, antioxidant, and plant growth–promoting activity (Figs. 4, 5, 6, 7, 8, 9; Table 1). This highlights significant strain-level variability, suggesting that genetic or metabolic differences may underlie the reduced activity observed in TP-BAC3. Recognizing such intra-species divergence is crucial for justifying strain-specific selection rather than assuming uniform functionality at the species level.

Moreover, *F. oxysporum* caused severe membrane damage (MII: 89% and MSI: 11%), consistent with its pathogenic role in disrupting cell integrity as reported previously (Mahmoud et al. 2021). All fungal treatments showed varying degrees of protection against Fusarium-induced membrane damage in *Phaseolus vulgaris*, as reflected by significant improvements in membrane stability index (MSI) and reductions in membrane injury index (MII). Among the isolates, *Talaromyces pinophilus* TP-BAC1 demonstrated the most marked effect, with the lowest MII (23.52%) and highest MSI (71.48%), indicating strong membrane protection, likely through enhanced antioxidant defense. *Trichoderma harzianum* and *Paceliomyces variotii* also significantly maintain membrane integrity, which is consistent with previous reports highlighting their roles in mitigating oxidative stress and lipid peroxidation under pathogen pressure (Ju et al. 2023; Kour et al. 2023). *Trichoderma longibrachiatum* and *Talaromyces pinophilus* TP-BAC2 showed moderate protective effects, while *Talaromyces pinophilus* TP-BAC3 and *Aspergillus fumigatus* showed limited improvement, with higher MII and lower MSI values. These differences suggest that the isolate varied capabilities for bioprotection, aligning with the findings that not all PGPF exert equal efficiency in maintaining membrane integrity under biotic stress (Anthony et al. 2017).

Oxidative stress triggered by *Fusarium* infection led to a dangerous disorder to plant cells and increased the levels of malondialdehyde (MDA) and hydrogen peroxide (H_2_O_2_) in the leaves of *P. vulgaris*. These results agreed with (Hasanuzzaman et al. 2021), in which the plants exposed to Fusarium infection showed highly increased MDA and H_2_O_2_ content in comparison to healthy control plants. Moreover, application of PGPF led to decrease in the content of H_2_O_2_ by 0.64%, 0.75%, 0.85%, 0.91% and 0.93%, and at* Talaromyces pinophilus* TP-BAC1,* Trichoderma longibrachiatum*,* Trichoderma harzianum, Paceliomyces variotii*, and *Talaromyces pinophilus* TP-BAC2, respectively and in the content of MDA by 0.12%, 0.15%, 0.8% and 0.23% at* Trichoderma harzianum, Talaromyces pinophilus* TP-BAC1,* Talaromyces pinophilus* TP-BAC2 and *Paceliomyces variotii* (Fig. 9 C), respectively compared to infected plants suggesting efficient ROS scavenging and improved systematic resistance. The high MDA in *F. oxysporum*-infected plants reflects oxidative damage. The obtained results are consistent with those reported in a previous study (Verma et al. 2022), which indicated that the utilization of biological stimulators under stressful circumstances resulted in a decrease of MDA and H_2_O_2_. These results correlate with studies showing that fungal endophytes mitigate biotic and abiotic stress (Gowtham et al. 2024). These results encourage the prospective application of nodule-associated fungal endophytes as biocontrol agents against *F. oxysporum.* Although some isolates showed similar antifungal activity in ethyl acetate assays, their disease suppression in greenhouse trials differed markedly. This suggests that direct antibiosis alone may not explain protection, and host-mediated responses, such as induced systemic resistance, likely play a key role, especially for TP-BAC1. Even if the greenhouse results were promising, field application may present additional challenges. Under natural conditions, introduced biocontrol agents must compete with the native soil microbiome and adapt to environmental variability, including fluctuations in temperature, moisture, and soil characteristics.

The two-way hierarchical clustering and heatmap analysis (Fig. 10) provide meaningful insights into the functional performance of the evaluated fungal treatments. Treatments grouped in Cluster II, including *T. pinophilus* TP-BAC1, *T. longibrachiatum*, *T. harzianum*, and *P. variotii*, were associated with high chlorophyll content, enhanced biomass accumulation, elevated phenolic and flavonoid levels, and reduced oxidative stress markers (H₂O₂, MDA)-patterns that correspond closely with their strong biocontrol efficacy and growth-promoting effects observed in greenhouse assays. Conversely, the pathogen-only treatment clustered separately due to its high oxidative stress indicators and poor physiological performance, validating the biological relevance of the clustering structure. These relationships demonstrate that the multivariate patterns not only differentiate treatments statistically but also reflect underlying mechanistic differences in plant protection and physiological enhancement driven by the fungal isolates. The accumulation of phenolics and flavonoids was strongly linked to improved growth in this study, corroborating an earlier study indicating secondary metabolites function as both antioxidants and growth-enhancing compounds in stressed plants (Wang et al. 2022).

**Fig. 11:**
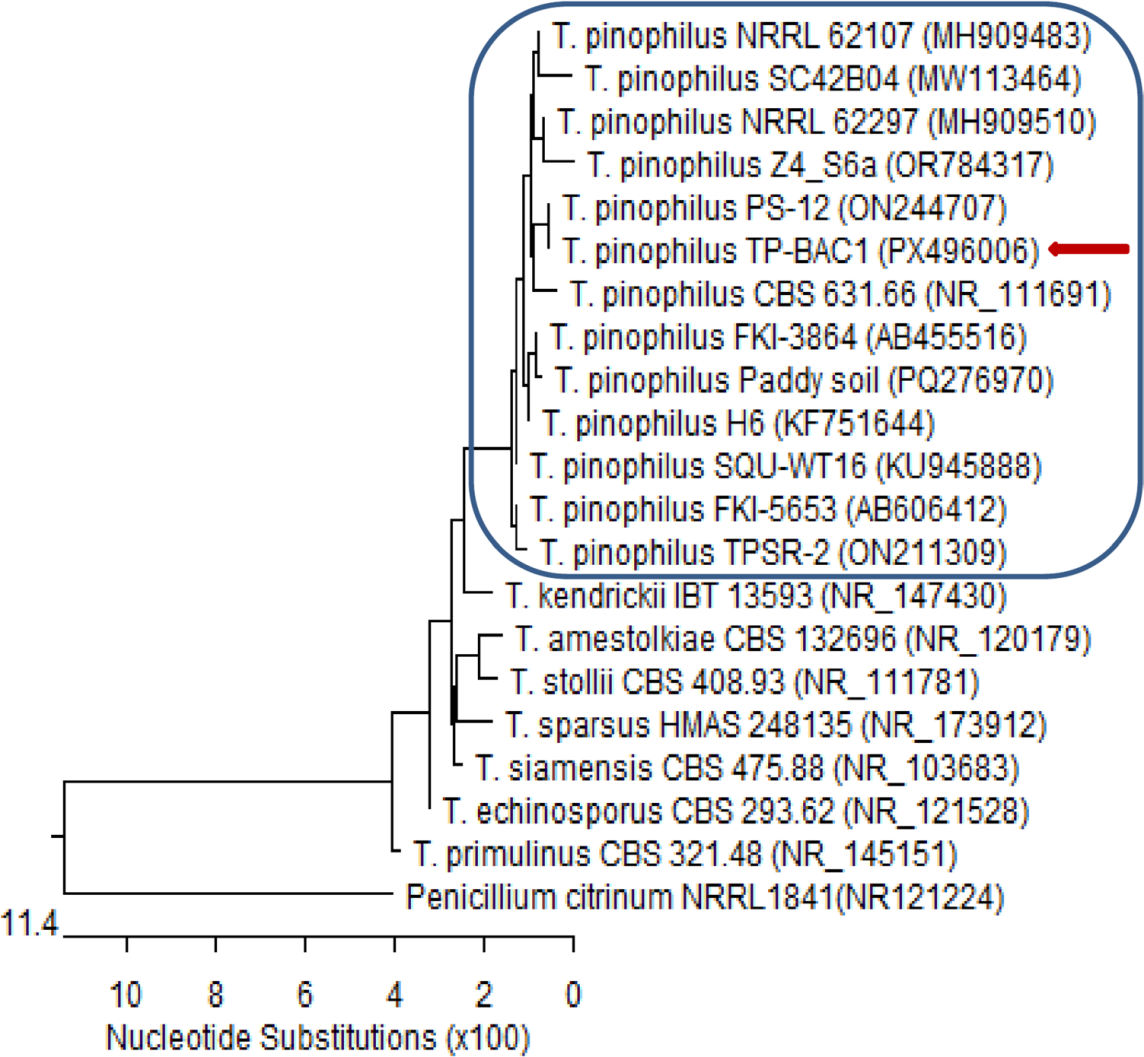
Phylogenetic tree based on ITS sequences of rDNA of the fungal strain isolated in the present study (*Talaromyces pinophilus* TP-BAC1 (accession: PX496006, arrowed) aligned with closely related strains accessed from the GenBank. This strain showed 99.20%—100% identity and 100% coverage with several strains of *T. pinophilus,* including the type material CBS 631.66 (accession: 111,691). *Penicillium citrinum* is included as an outgroup strain. [T. = *Talaromyces*]

**Fig. 12:**
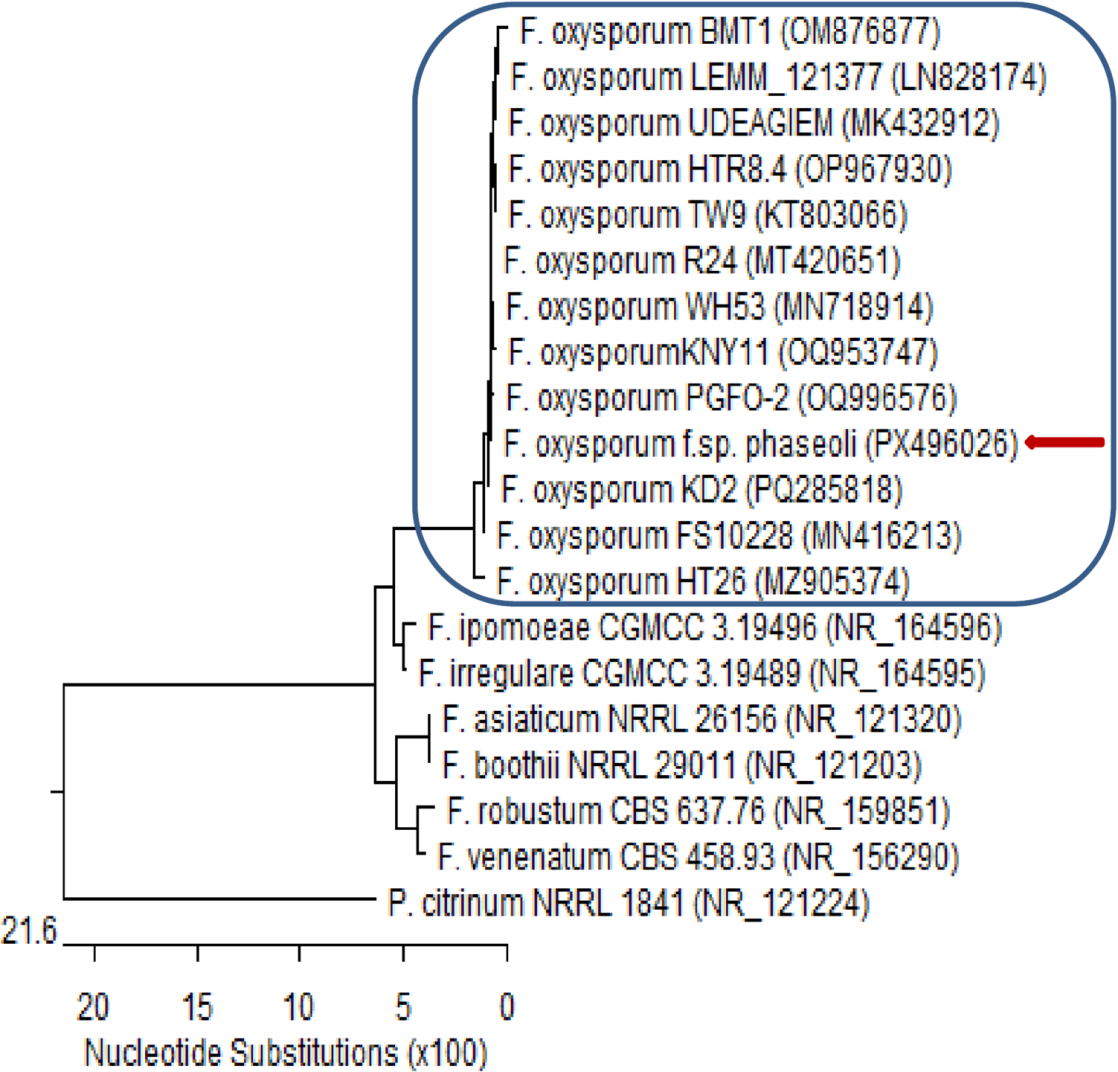
Phylogenetic tree based on ITS sequences of rDNA of the fungal strain isolated in the present study (*Fusarium oxysporum* f. sp. *phaseoli* (accession: PX496026), arrowed) aligned with closely related strains accessed from the GenBank. This strain showed 100% identity and 100% coverage with several strains of *F. oxysporum*. *Penicillium citrinum* is included as an outgroup strain. [F. = *Fusarium*, P. = *Penicillium*]

Although the greenhouse assays demonstrated the strong biocontrol potential of *Talaromyces pinophilus* TP-BAC1 and other nodule-associated fungi, translating these results to field conditions presents additional challenges. Soil microbial communities in natural environments are far more complex, and introduced endophytes must compete with established native microbiota for space and resources. Moreover, environmental variability-including fluctuations in soil moisture, temperature, pH, and nutrient availability-may influence colonization efficiency, metabolite production, and overall biocontrol performance. These factors can affect the consistency of fungal activity compared to controlled greenhouse settings. Therefore, while our results are promising, field-scale validation is essential to confirm the robustness and practical applicability of these isolates under diverse agricultural conditions (Figs. 11 and 12).

## Conclusion

The current study provided strong proof that nodule-associated fungi can fulfill a dual role in bean cultivation. They exhibited considerable antifungal efficacy against *F. oxysporum* f. sp. *phaseoli*, the pathogen responsible for Fusarium wilt, a severely detrimental soil-borne disease affecting legumes. Conversely, they significantly enhanced the growth and vitality of bean plants in greenhouse conditions. This dual effect reduces disease severity while improving overall plant performance, highlighting their function as both growth-promoting and disease-managing agents in succession. NBF's ability to stimulate host immunity suggests the existence of complex resistance mechanisms, offering a significant potential for further investigation. Moreover, confirmation of nitrogen-fixing activity requires targeted quantitative or molecular assays. These findings highlight the potential of NBF as sustainable, eco-friendly biocontrol agents that could reduce reliance on chemical fungicides and improve agricultural ecosystems.

## Data Availability

Data is provided within the manuscript or supplementary information files.

## Abbreviations

CAS: Chrome azurol S
DMSO: Dimethyl sulfoxide
DPPH: 2,2-Diphenyl-1-picrylhydrazyl
DS: Disease severity
EC: Electrical conductivity
EtOAc: Ethyl acetate
Fop: *Fusarium oxysporum* F. sp. *phaseoli*
GA_3_: Gibberellic Acid
GAEs: Gallic acid equivalents
H₂O₂: Hydrogen peroxide
IAA: Indole acetic acid
MDA: Malondialdehyde
MII: Membrane Injury Index
MSA: Mineral Salt Agar
MSI: Membrane stability Index
NBF: Nodule borne fungi
NBRIP: National Botanical Research Institute's Phosphate
NREF: Non-rhizobial endophytic fungi
PCA: Principle component analysis
PDA: Potato dextrose agar
PDB: Potato dextrose broth
PDI: Percent disease index
PGP: Plant growth-promoting
PGPF: Plant growth promoting fungi
QE: Quercetin
TBA: Thiobarbituric acid
TC: Total count
TCA: Trichloroacetic acid
